# An Integrative Method for Accurate Comparative Genome Mapping

**DOI:** 10.1371/journal.pcbi.0020075

**Published:** 2006-08-04

**Authors:** Firas Swidan, Eduardo P. C Rocha, Michael Shmoish, Ron Y Pinter

**Affiliations:** 1 Department of Computer Science, Technion, Israel Institute of Technology, Haifa, Israel; 2 Janelia Farm Research Center, Howard Hughes Medical Institute, Ashburn, Virginia, United States of America; 3 Atelier de Bioinformatique, University Paris VI, Paris, France; 4 Unité GGB, Institut Pasteur, Paris, France; University of California San Diego, United States of America

## Abstract

We present MAGIC, an integrative and accurate method for comparative genome mapping. Our method consists of two phases: *preprocessing* for identifying “maximal similar segments,” and *mapping* for clustering and classifying these segments. MAGIC's main novelty lies in its biologically intuitive clustering approach, which aims towards both calculating reorder-free segments and identifying orthologous segments. In the process, MAGIC efficiently handles ambiguities resulting from duplications that occurred before the speciation of the considered organisms from their most recent common ancestor. We demonstrate both MAGIC's robustness and scalability: the former is asserted with respect to its initial input and with respect to its parameters' values. The latter is asserted by applying MAGIC to distantly related organisms and to large genomes. We compare MAGIC to other comparative mapping methods and provide detailed analysis of the differences between them. Our improvements allow a comprehensive study of the diversity of genetic repertoires resulting from large-scale mutations, such as indels and duplications, including explicitly transposable and phagic elements. The strength of our method is demonstrated by detailed statistics computed for each type of these large-scale mutations. MAGIC enabled us to conduct a comprehensive analysis of the different forces shaping prokaryotic genomes from different clades, and to quantify the importance of novel gene content introduced by horizontal gene transfer relative to gene duplication in bacterial genome evolution. We use these results to investigate the breakpoint distribution in several prokaryotic genomes.

## Synopsis

Comparative genomics is an important discipline with applications in evolutionary, genetic, and genome rearrangement studies. When comparing genomes, one is usually interested in investigating the relation between the genomic segments to establish their evolutionary origin: are the segments orthologous, and hence inherited from their most recent common ancestor? Are they paralogs, and hence duplicated from an ancestral segment? Did the segments undergo reordering? Were the segments deleted or inserted and—if so—how (insertion sequence, prophage, horizontal gene transfer)?

In this paper, Swidan et al. present MAGIC, a new approach for comparative genome mapping. The main novelty of this approach is the biologically intuitive clustering step, which aims towards both calculating reorder-free segments and identifying orthologous segments. The authors demonstrate MAGIC's robustness, relative to both its initial input and to its parameters' values. MAGIC's scalability is demonstrated by running it on distantly related organisms and on large genomes. In addition, Swidan et al. provide a detailed analysis of the differences between MAGIC and other comparative mapping methods.

Applying MAGIC to several prokaryotic pairs enabled the authors to address the aforementioned questions and to quantitatively study the different evolutionary forces shaping the prokaryotic genome as well as to investigate their breakpoint distribution.

## Introduction

In the context of *comparative genome mapping,* one seeks to identify “homologous” segments in different genomes and to classify them into orthologs and paralogs, as well as to identify segments “free of reordering.” Segments belonging to different genomes are said to be *homologous* if they descend from a single common ancestral segment [1–3]. Segments belonging to different genomes are said to be *free of reordering* or *reorder free* (RF) if they were not reordered in the different genomes relative to their *cenancestor,* i.e., their most recent common ancestor [2]. RF segments may contain large indels, resulting, for example, from deletions, duplications, horizontal gene transfer (HGT), or selfish DNA. Thus, in the context of comparative genome mapping, one attempts to achieve a detailed description of the common and different parts between whole genomes with respect to large-scale mutations. This description, along with a characterization of point mutations in homologous segments, enables us to study the relative frequency and the effect of the different types of mutations (or forces) shaping the evolution of the genomes. Since the biological events considered in the comparative mapping problem are substantially different from those considered in the alignment problem, neither global alignment nor local alignment techniques are sufficient to address it.

Most of the pioneering studies considered the problem of comparative mapping over sets of genes instead of arbitrary genomic segments. These methods start, usually, by calculating an all-against-all alignment of common sets of genes (a preprocessing phase), and then, in a second phase, use clustering techniques to predict operons or collinear blocks. Such approaches include Lamarck, the P-quasi complete linkage approach, ADHoRe, EM_TRAILS, STRING, and other methods [4–10]. This approach was also applied in yeast for the automatic discovery of regulatory motifs [11]. Later, comparative mapping methods over arbitrary genomic segments were developed. The preprocessing phase in these methods consists of searching for similar genomic segments—referred to as *hits, markers,* or *anchors* (usually performed by a fast local alignment procedure). Then, in the mapping phase, a clustering procedure is applied to the output of the first phase. Examples of fast seed-based preprocessing phases include BLASTZ and CHAOS [12,13], in which the seeds are allowed to contain degeneracy, as well as that of Mauve, which searches for exact and unique matches [14]. Examples for mapping phases include CHAIN-NET, FISH, GRIMM-Synteny, Mauve (as well as GRIL—its predecessor), and SLAGAN [13–18]; for a mini-review see [19]. CHAIN-NET and GRIMM-Synteny use the distance between anchors as a criterion for clustering (and hence are referred to as *distance-based* mapping methods). Mauve and SLAGAN rely on solving (different) optimization problems to prune anchors and to define the mapping. SLAGAN prunes anchors by introducing various gap penalties for discriminating between different subsets. Finally, FISH is based on a statistical model for anchor clustering.

**Figure 1:**
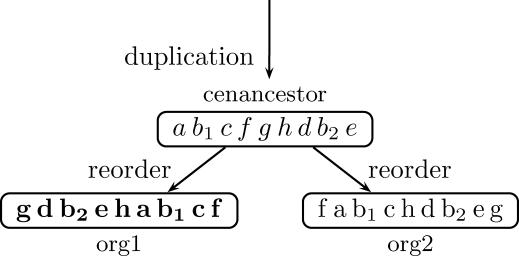
A Hypothetical Example Demonstrating the Definition of Positional Orthologs and the Emergence of a Nuisance Cross-Overlap A portion of the genomic segments in a hypothetical cenancestor is denoted by letters. Their orthologous segments in the descendant organisms org1 and org2 are given, using the same letters, but in different font (to stress that the segments, despite being orthologous, are similar but not identical). The scenario described in this example is as follows: a duplication of a genomic segment results in two duplicates *b*_1_ and *b*_2_ in the cenancestor. During the speciation of org1 and org2 the cenancestor genomic segments are shuffled. The orthologous segments **b1** and b1 have similar genomic contexts and are thus positional orthologs. Similary **b2** and b2 are positional orthologs as well. When comparatively mapping org1 and org2, one would find that **b1** is similar to b2 and **b2** is similar to b1. These hits obscure the deduction of the true evolutionary relation between **b1** and b1 as well as between **b2** and b2, and are referred to as nuisance cross-overlaps. In real biological examples, similar situations arise, e.g., because of rDNAs; see Figure 2. Notice also that, unlike in sequence alignment, and as is demonstrated in this example, duplications that occurred *before* the cenancestor (referred to sometimes as *outparalogs* [3]) may cause hardships when comparatively mapping two organisms. Thus, nuisance cross-overlaps can be thought of as an “ancestral curse.”

**Figure 2:**
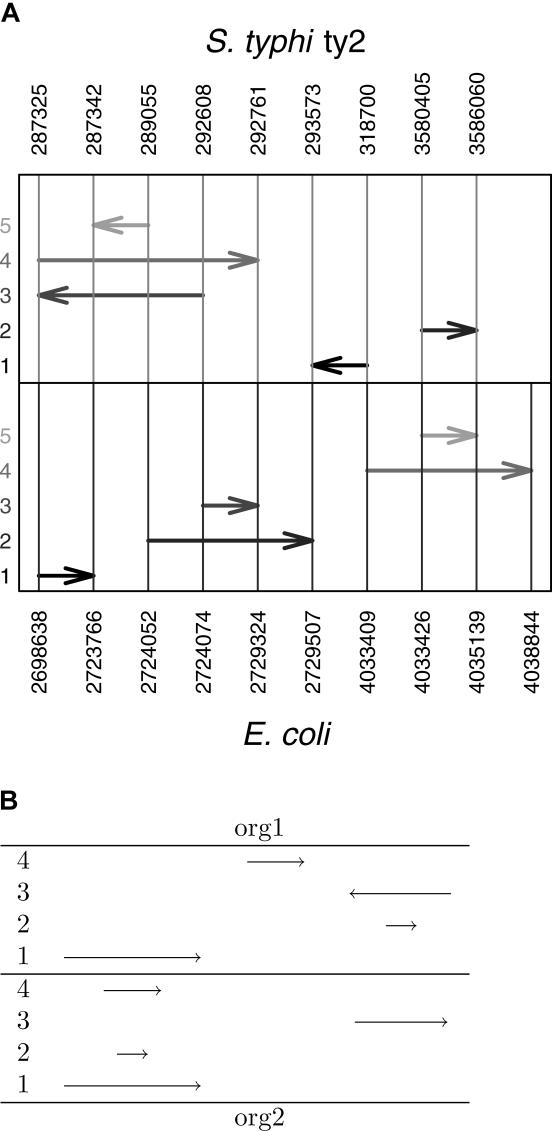
Schematic Examples of Situations That May Be Encountered While Clustering Entries of the Comprehensive Table (Steps 2 and 3 in the Mapping Phase) An entry is represented as two arrows having the same label, one in the top and one in the bottom panels. Labels for the arrows are given on the left side. The relative direction of identically labeled arrows represents the relative orientation of the corresponding segments. (A) A real example from the comparison of E. coli versus S. typhi ty2 demonstrating the difficulty of identifying the consecutive entry. In the top (bottom) panels, the positions of the segments in S. typhi ty2 (E. coli) are given. Arrows having the same label are drawn with identical grayscale color. The significantly overlapping entries, e.g*.*, 2 and 3 in *E. coli,* correspond to rDNA operons, of which there are seven units in each organism. Each of these seven units have a hit overlapping, for example Entry 2 in both top and bottom panels. For the sake of clarity, the figure demonstrates only a part of the hits and is schematic (not to scale). (B) Illustration of nuisance cross-overlaps and inparalogs. Entry 2 overlaps with Entry 1 in org1 and with Entry 3 in org2. Assuming that both overlaps are long enough and that Entry 2 is significantly shorter than either Entry 1 or Entry 3, Entry 2 is considered as a nuisance cross-overlap. On the other hand, Entry 4 overlaps Entry 1 in org2, but does not overlap with other entries in org1, and hence is not a nuisance cross-overlap. Assuming the length of Entry 1 is significantly greater than that of Entry 4, Entry 1 is considered to be the positional ortholog, while Entry 4 is considered to be the inparalog.

Here we present MAGIC, an integrative and accurate tool for comparative genome mapping. MAGIC consists of two independent phases: a preprocessing phase, in which we compute a *comprehensive* table of all *maximal similar segments* (see Preprocessing Phase: Building a Comprehensive Table of Similar Segments), and a mapping phase for clustering the table into RF regions. MAGIC's clustering approach is based on a new definition of “consecutive homologous segments” which relies on a biologically intuitive ordering of similar segments (see Mapping Phase: Clustering into RF Segments). It enables a better handling of duplications in general and allows us to adequately address the problem of “nuisance cross-overlaps,” i.e*.*, misleading similarities between duplications that occurred before the most recent common ancestor (see Figures 1 and 2 for examples). Nuisance cross-overlaps can introduce significant artifacts in the mapping (see False Anchors and A Comparison with Mauve's Results for examples), and, to the best of our knowledge, were not taken explicitly into account before. MAGIC is also robust with respect to both its parameters' values and the initial set of anchors (see MAGIC's Robustness). It is capable of modifying and refining the mapping induced from the anchors and even recognizing and reassigning false orthologs in the initial anchor set itself (see False Anchors). Furthermore, MAGIC is scalable and can be applied to distantly related pairs and to large genomes (see MAGIC's Scalability). Finally, our approach is explicitly designed to handle circular genomes (by considering the last and first nucleotides to be successive). The output of our algorithm consists of detailed coverage statistics of the genomes and of several tables including a one-to-one table describing the RF segments.

We have applied our method to several prokaryotic pairs spanning different branches of the tree of life. Due to the quality of their sequences, annotations, and the availability of many closely related sequenced species, prokaryotic genomes are very good models to test the quality of the mapping. MAGIC's analysis of the different forces shaping these genomes shows that lateral transfer and large deletions affect them significantly more than duplications (see Major Forces Shaping the Prokaryotic Genome). Furthermore, we utilized MAGIC's results to analyze the breakpoint distribution in bacteria. Previous studies, on Salmonella typhimurium [20,21] and on Escherichia coli [22,23], have shown that they contain noninvertible (or nonpermissive) segments. For some of these segments, forcing an inversion by mechanisms different from those found in the cell resulted in organisms that are viable and that grow normally [24]. Thus, these studies suggest that the mechanisms of reordering in these bacteria may inherently cause deviations from a uniform breakpoint distribution [20]. In addition, reorderings in bacterial genomes are constrained by the viability and fitness of the resulting organisms. These selection constraints result in operons and in the large-scale organization of the genome relative to replication [25]. Finally, repeats, a major element of genome disruptions caused by homologous recombination [26], are by themselves recombination hotspots [25,27]. Surprisingly, however, statistical tests show that, in many pairs, the breakpoint distribution fits well to the uniform distribution (see Breakpoint Distribution section). This controversy extends the debate about the Nadeau-Taylor model and the existence of hotspots in mammalian genomes to prokaryotes [17,28–35].

To relate our method to previous work, we give a detailed comparison between MAGIC's results and that of other well-known genome mapping tools (see A Comparison with Mauve's Procedure and A Comparison with Mauve's Results).

A C++ implementation of MAGIC and a Java-based graphical user interface (GUI) are under development. They will be made available at http://magicmapping.sourceforge.net.

### Terms and Definitions

The relation of being orthologous can be further refined to “positional orthology”—see Figure 1. Segments belonging to different genomes are said to be *positional orthologs* if they are orthologs and have preserved their relative positioning or genomic contexts in the genomes. The related term “positional homologs” was presented in [36,37] to refine the homology relation. The paralogy relation can be further refined to “outparalogs” and “inparalogs” [3]: a segment that has duplicated before (after) the speciation from the cenancestor is referred to as an *outparalog* (*inparalog*). Outparalogs induce the phenomenon of nuisance cross-overlaps, complicating the comparative mapping (see Figure 1). Identifying inparalogs, on the other hand, is required to quantify the amount of duplications that occurred since the divergence of the taxa.

## Results/Discussion

**Figure 3:**
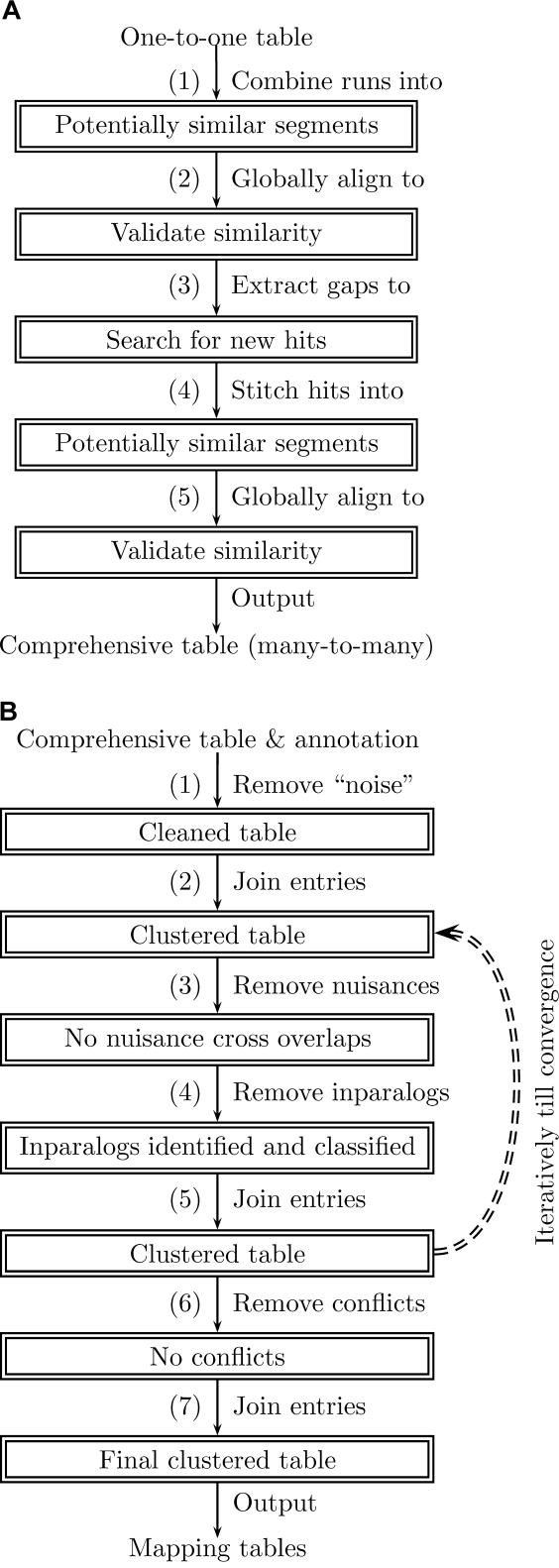
A Flow Diagram of MAGIC (A) A flow diagram of the preprocessing phase (see Preprocessing Phase: Building a Comprehensive Table of Similar Segments). (A1) A one-to-one table (anchors) between two genomes is used to calculate runs of anchors corresponding to potentially similar segments. (A2) These segments are globally aligned to validate their similarity and to find unmatched regions (these regions are candidates for indels or reordering events). (A3) The unmatched regions are extracted (the entries remaining in the table correspond thus to maximal similar segments) and aligned (including uncovered regions between the runs) locally against the other genome to search for new hits, in an attempt to make the table comprehensive. (A4) Local hits are stitched together to form potentially similar segments. (A5) These segments are globally aligned to validate their similarity and to extract unmatched regions out of them. The resulting table is comprehensive and consists of maximal similar segments. (B) A flow diagram of the mapping phase (see Mapping Phase: Clustering into RF Segments). (B1) Short entries and entries corresponding to known selfish DNA in either of the genomes are removed. (B2) Consecutive entries are joined for the first time (which makes it easier to identify nuisance cross-overlaps and inparalogs). (B3) Nuisance cross-overlaps (see Steps 3–5: Identifying nuisance, classifying inparalogs, and re-clustering and Figure 2) are identified and discarded. (B4) Inparalogs are identified, classified, and removed from the table. (B5) Consecutive entries are joined again (nuisances and inparalogs may have hindered joining some of the entries in the previous clustering). Steps B3–B5 are performed iteratively until the table converges (no more nuisance or inparalogs are identified). (B6) Remaining conflicts (unresolved significant overlaps), corresponding most likely to selfish DNA, are removed. (B7) Consecutive entries are joined for the final time (the conflicts removed in B6 may have hindered joining some of the entries in the previous clustering). The output of the mapping phase consists of several tables, among which are: a one-to-one table describing the RF segments, a one-to-one table describing the positional orthologous segments contained in the RF segments, a table of the classified inparalogs in each organism, and a table of identified transposable elements.

MAGIC's preprocessing phase constitutes a linear flow of global and local alignments and can be described as a cascade of five steps (Figure 3A). We first choose a set of anchors, which are joined into consecutive runs to serve as a first (likely inaccurate) table. Any set of anchors can be chosen. In the present analysis, we used the list of curated orthologs of KEGG (KO) [38], since it is based on functional and positional information [5], in addition to sequence similarity. Note, however, that the anchor set can be derived from other sources. For example, in the comparison with other comparative mapping methods, we have used their automatically generated output as a set of anchors (see A Comparison with Mauve's Results). The table is then refined by validating the similarity of existing correspondences (by global alignment) and extracting unmatched regions. This is followed by looking for new hits in the unmatched regions (by local alignment). Because of MAGIC's ability to discover and reassign “false” anchors (see False Anchors) and its robustness with respect to the initial set of anchors (see MAGIC'S Robustness), it is suitable to be used with any set of (noisy) anchors.

In the mapping phase (Figure 3B), the comprehensive table resulting from the preprocessing phase is cleaned from short entries and selfish DNA. Then a clustering technique is applied to the remaining entries to combine RF segments, determine positional orthologs, and classify inparalogs.

The design of the method aims towards maximizing the flexibility of replacing existing components with new ones as soon as those become available. Thus, for example, the anchor set can be chosen arbitrarily. Also, the local or global alignment programs from the preprocessing phase can be readily replaced. Eventually, the whole preprocessing phase can be replaced with another one.

In the following we present a description of the two phases; further details can be found in Materials and Methods, as noted throughout. In the section Preprocessing Phase: Building a Comprehensive Table of Similar Segments, we describe the steps involved in the preprocessing phase (building the comprehensive table). Then, in Mapping Phase: Clustering into RF Segments, we describe the mapping phase. A summary of MAGIC's parameters is given in Table 1. Tuning these parameters is discussed in Tuning the Parameters. Finally, in A Comparison with Mauve's Procedure, we compare our method to previous work. While some of the algorithms are based on existing tools (the dependencies are noted in the text), the rest was implemented as R [39] and BASH scripts (a C++ implementation with a Java-based GUI are under development).

**Table 1:**
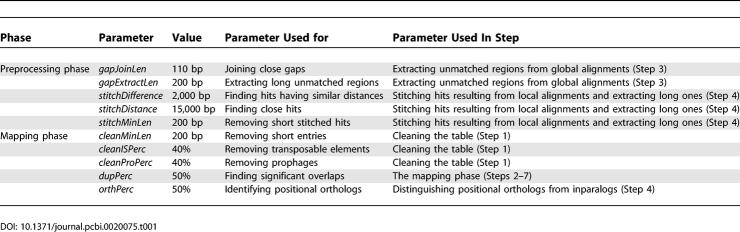
A Summary of the Parameters Used in MAGIC

### Preprocessing Phase: Building a Comprehensive Table of Similar Segments

Here we describe Steps 1–5 of the preprocessing phase (see Figure 3A) in detail. Some of our steps require a more complicated mathematical formalism so as to be well-defined. For these, the formal definitions are given in Materials and Methods.

#### Step 1: Generating runs of anchors.

**Figure 4:**
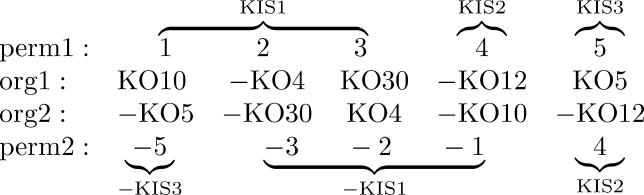
Renaming Common Anchors in Genomes org1 and org2 to Permutations perm1 and perm2 (Step 1 of the Preprocessing Phase) The genome org1 is renamed to the identity permutation (perm1) and the genome org2 is renamed accordingly. Runs of consecutive numbers in perm2 are combined (with respect to their signs) into KISs. A negative sign preceding a KO indicates that the KO is coded on the complementary strand. A negative sign preceding a permutation element indicates that its orientation is not identical in both genomes.

To generate a first coarse mapping, we join consecutive anchors into runs: given two genomes, we generate *permutations* of signed elements [17] based on their common unique anchors, e.g., KOs—see Figure 4. The signs represent the relative orientation of the KOs in the two genomes. The two permutations are renamed so that one becomes the identity permutation (with positive elements). The other permutation is then searched for maximal runs of consecutive numbers (while taking their signs into account). We refer to a genomic segment corresponding to such a run as a *KO-induced segment* (KIS). The procedure is illustrated in Figure 4.

#### Step 2: Global alignment of KISs.

To validate the similarity of the KISs in the two organisms, we globally align them. Among the currently available methods for global alignments, e.g., [40–49] (see also [50] for a recent review), we used AVID [41]. AVID is a heuristic that runs in time and space that are linear with respect to the size of the genomic sequences. Still, to cope with memory limitations, KISs longer than 200,000 bp are divided into subsegments such that successive pieces start and end with the same KO.

#### Step 3: Extracting unmatched regions and local alignments.

**Figure 5:**
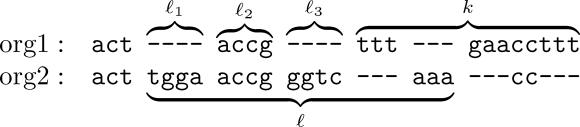
Joining Gaps into Unmatched Regions and Extracting Long Ones from a Sequence Alignment (Step 3 of the Preprocessing Phase) Gaps *ℓ*_1_ and *ℓ*_3_ in org1 are joined if the (intragapped) hit *ℓ*_2_ is short enough. Assume that *ℓ*_2_ and other intra-gapped regions are short enough so that the proximal gaps in org1 are joined to form the unmatched region *ℓ*. Assume that gaps in org2 are joined similarly to form the unmatched region *k,* and that the unmatched regions *ℓ* and *k* are long enough. If the regions *ℓ* and *k* intersect (as in this example), they are joined and the resulting segments in both organisms are extracted to be handled in the next step.

To identify unmatched regions in the global alignment, we group proximal gaps and extract sufficiently long ones—see Figure 5. The unmatched regions are either segments that have undergone reordering (and thus disturb the collinear global alignment) or indels. To distinguish between the two cases, we search the other genome for hits based on these regions. If hits are found, the unmatched regions might result from reordering mutations (this is determined in the mapping phase—see Mapping Phase: Clustering into RF Segments), or otherwise they are most likely indels. The unmatched regions are constructed by joining close gaps in each of the two genomes separately (*<gapJoinLen* parameter). Afterwards, sufficiently long unmatched regions (*>gapExtractLen* parameter) that overlap are merged together, the merged unmatched regions are extracted, and the alignment is broken at the corresponding points. The extracted unmatched regions, along with the uncovered regions between KISs, are locally aligned against the other genome. Among the currently available methods for local alignment, e.g., [12,43,51–53], we used BLAST [54]. Tuning the parameters is discussed under Tuning the Parameters. Pseudo-code for the procedure is given in Algorithm 1, in Materials and Methods.

#### Step 4: Stitching local matches.

**Figure 6:**
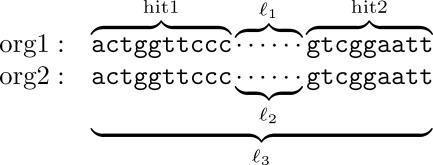
Parameters Considered When Stitching Hits Having the Same Orientation (Step 4 of the Preprocessing Phase) To stitch the hits hit1 and hit2, we consider first the distance between them in both organisms (length *ℓ*_1_ and *ℓ*_2_, respectively). If the segments corresponding to the lengths *ℓ*_1_ and *ℓ*_2_ are similar, then *ℓ*_1_ and *ℓ*_2_ are expected to have similar values. Assuming the latter, the difference between the distances of the two hits, i.e., |*ℓ*_1_ − *ℓ*_2_ |, should be small. In addition, consecutive hits in similar segments are expected to be close, i.e., both distances, *ℓ*_1_ and *ℓ*_2_, should be small. Finally, if hit1 and hit2 are stitched (with no other hits stitched to them), we check that the total segment's length in each organism is long enough. In org2 this length is referred to as *ℓ*_3_.

To calculate new potential maximal similar segments, each set of local matches is scanned for hits that can be stitched together. Stitched hits need to have the same orientation. To determine which hits to stitch, three quantities are considered—see Figure 6. The first is the difference between the *distances* of the two hits in the two organisms (|*ℓ*_1_ − *ℓ*_2_| in Figure 6). Intuitively, the distance between two hits in a given organism is calculated by subtracting the end of the one hit from the start of the other—see the section, Formal description of stitching local matches, for the exact definition and Figure 6 for an illustration. If the segments between the two hits are similar, the distances between the two hits in the two organisms should be similar. Therefore, hits with an excessive difference in their distances (*>stitchDifference* parameter) are not stitched. Second, we consider the distance between the two hits (*ℓ*_1_ and *ℓ*_2_ in Figure 6). If two segments in the two organisms are similar, one would expect to find many hits when locally aligning them. Thus, the distance between consecutive hits in similar segments should be short. Therefore, hits with too large a distance (*>stitchDistance* parameter) are not stitched. Finally, after stitching hits that fulfill the above two requirements, we keep stitched segments (*ℓ*_3_ in Figure 6) that are long enough (*>stitchMinLen* parameter). Tuning the parameters is discussed in Tuning the Parameters. A formal definition of the quantities considered in this step is included in the section Formal description of stitching local matches. Pseudo-code for the procedure is given in Algorithm 2, in Materials and Methods.

#### Step 5: Global alignment of new segments.

Potentially similar segments that were stitched together are globally aligned to validate their similarity, and unmatched regions are extracted from them. However, unlike the previous round of global alignments, this time there is no need to locally align the unmatched regions against the other genome—they cannot have significant hits since they were not picked up by the previous local and global alignment steps.

### Mapping Phase: Clustering into RF Segments

At this point we have a comprehensive table of maximal similar segments between the two organisms. However, because of repeated elements, e.g*.*, duplicated rDNA operons and ISs, the mapping is not one-to-one. Moreover, due to insertions, deletions, and duplications, consecutive elements belonging to the same RF segment are hard to identify. In the remainder of this section, we present a novel approach for defining consecutive entries that is capable of handling duplications in general, and nuisance cross-overlaps in particular. A flow diagram summarizing the mapping phase as a cascade of seven steps is given in Figure 3B.

#### Step 1: Removing trivial entries and selfish DNA.

We start with the comprehensive table that was created in the preprocessing phase. First we remove short entries (*<cleanMinLen*). Then we identify and remove transposable elements and prophages (when applicable). These elements replicate and integrate autonomously in the genome [55]. Therefore, unless discarded before constructing the RF segments, they can lead to wrong assumptions about genome dynamics and an incorrect mapping. The cleaning is done by first identifying transposable elements in both genomes. Then, entries in the table having too long an intersection (*>cleanISPerc*) with the identified transposable elements are discarded. Since sequences of transposable elements diverge fast, we used the tblastx mode of BLAT [52] to identify them. To do the search, a database of all known transposable elements in bacteria was obtained through SRS [56] on EMBL [57].

Prophages, on the other hand, are harder to identify because their sequences diverge fast, they often have genes that are similar to chromosomal genes, and both functional and remnant prophages (e.g., cryptic and mosaic) often have only residual similarity with other known functional phages [58]. Guidelines for identifying prophages were introduced in [58] and were implemented in [59]. We use the latter annotation for discarding entries in the table intersecting with prophages in a similar manner to transposable elements. We refer to the threshold used for the prophages as c*leanProPerc*.

Finally, we join entries that overlap in both organisms and move toward constructing the RF segments.

We emphasize that MAGIC can be run without the information on transposable elements or prophages (see, for example, the *Bordetella* pair in Table 2). However, keeping these elements is expected to result in a less accurate map with higher fragmentation (see, for example, A Comparison with Mauve's Results as well as Tables 3 and 4).

**Table 2:**
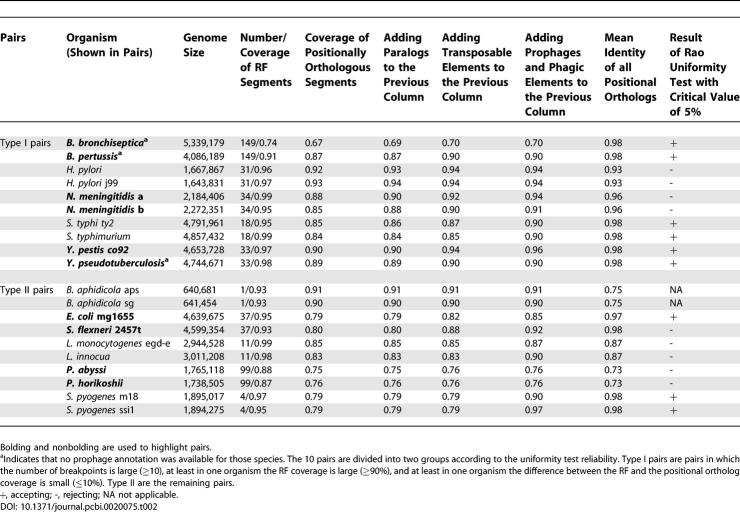
Pairwise Comparison Statistics

**Table 3:**
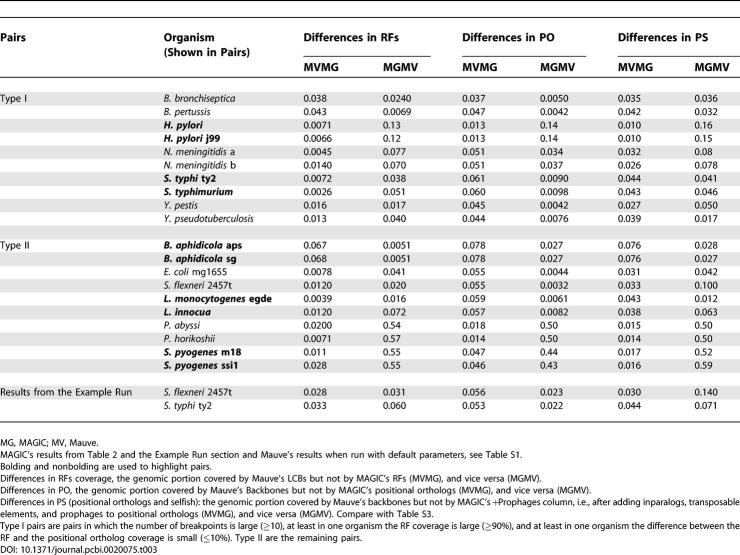
Comparing Coverage between MAGIC's Results and Mauve's Results When Run with Default Parameters

**Table 4:**
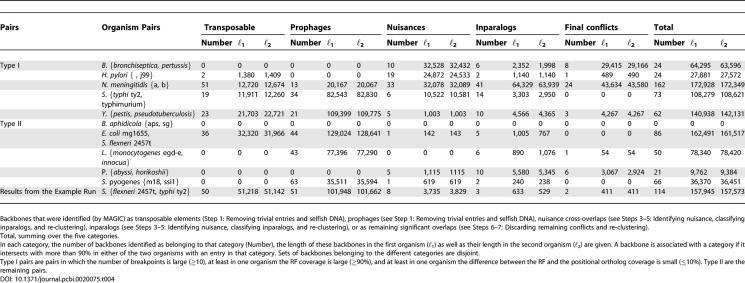
Running MAGIC with Mauve's Backbones as Anchors and Classifying these Anchors into Five Categories

#### Step 2: Finding consecutive entries and clustering into RF segments.

Duplications are one of the major hurdles when constructing RF segments because they introduce ambiguity that makes identifying consecutive entries hard. Figure 2A illustrates this difficulty by an example from the comparison of Escherichia coli K-12 MG1655 *(E. coli)* [60] versus Salmonella enterica serovar *typhi* ty2 (S. typhi ty2) [61]. The question that we want to answer is: which of the entries {2,…,5} from Figure 2A is consecutive to 1 (if any)? Intuitively, Entry 3 is the “natural” choice, since among all the entries that have the same orientation as Entry 1 in both organisms, Entry 3 is the “closest” to Entry 1 in both organisms. In the following we present a description for a method to generalize the above example. The description is intuitive and is demonstrated on Entries 1 and 3 from Figure 2A. The formal definitions are given in Formal description of clustering into RF segments.

Consider all the “significant overlaps” of Entries 1 and 3 in both organisms. Two entries are said to *overlap significantly* in a given organism if the percentage of their intersecting length in that organism relative to their length is large enough (*>dupPerc*). For example, Entry 2 overlaps significantly with Entry 3 in E. coli, while Entries 4 and 5 overlap significantly with Entry 3 in S. typhi ty2. Entry 1 does not overlap significantly with other entries in either of the organisms. Notice that even though Entries 1 and 3 are not successive in either of the organisms, Entry 2, which overlaps significantly with Entry 3 in *E. coli,* is successive to Entry 1 in E. coli. Furthermore, Entry 4, which overlaps significantly with Entry 3 in S. typhi ty2, precedes Entry 1 in S. typhi ty2. Thus, even though 1 and 3 are not successive in either of the organisms, Entry 3 overlaps significantly with entries in both organisms that are the closest to Entry 1. In addition, as mentioned earlier, Entries 1 and 3 have the same sign. Therefore, they are potentially a consecutive pair. Since this is the only potentially consecutive pair involving either Entry 1 or 3, these two Entries are considered to be consecutive. In general there might be another entry besides 3 that is potentially consecutive with Entry 1. In this case, we choose the entry that is closest to 1 (for more details, see the section Formal description of clustering into RF segments). After calculating the consecutive entry of each given one, we join consecutive entries together into initial RFs (Algorithm 3 in Materials and Methods).

#### Steps 3–5: Identifying nuisance, classifying inparalogs, and re-clustering.

The table resulting from the above clustering step would usually contain overlapping entries. To identify duplications correctly, we need first to identify and remove “nuisance cross-overlaps,” which are entries that overlap significantly with different initial RF segments in the different organisms. Figures 1 and 2B illustrate the definition. Nuisance cross-overlaps correspond to “fake” hits, e.g., low similarity segments or duplications that occurred before the most recent common ancestor (outparalogs), and need therefore to be discarded (see A Comparison with Mauve's Results for the artifacts that nuisance cross-overlaps can introduce in the mappings).

For the remaining significant overlaps, we consider those for which positional orthologs and inparalogs can be determined. Given two entries that overlap significantly according to one of the organisms, if one entry is at least *orthPerc* longer than the other, the former is considered to be the positional ortholog, and the latter—the inparalog. Figure 2B illustrates these definitions. Notice that the duplications that are considered in this method are those that happened in either of the two organisms since their divergence (inparalogs). Therefore, old duplications (outparalogs), e.g., the rDNA operons, are not relevant. This behavior is assured by removing the nuisance cross-overlaps. The set of inparalogs in each organism is further classified to identify segments that have multiple duplications. In addition, entries in the set of inparalogs that correspond to a single duplicated segment in one organism are identified and grouped together. Such entries might result in multiple counting of the same duplicated segment, unless counted as a single group.

After discarding the inparalogs, we extend the initial RF segments by calculating consecutive entries and joining them (by running Algorithm 3 in Materials and Methods) again. Steps 3–5 are performed iteratively until the table converges and no more nuisances or inparalogs are identified.

#### Steps 6–7: Discarding remaining conflicts and re-clustering.

Any significant overlaps that remain at this point correspond to isolated segments for which positional orthologs and inparalogs cannot be inferred—see Figure S1. Such entries are referred to as *conflicts*. Conflicts could correspond to unidentified selfish DNA segments and are thus discarded. Afterwards, we calculate consecutive entries and join them (by running Algorithm 3 in Materials and Methods) for the final time, which results in the final RF segments.

#### Comparison statistics.

To check the similarity between the two genomes, we calculate at this point the similarity between all the initial entries that were joined to construct the RF segments. This calculation is done by globally aligning these entries one more time. Based on the alignments, a weighted mean of their identity percentage is calculated for each genome, where the weights are the respective lengths of the entries in each organism. Furthermore, these entries, the RF segments, the classified inparalogs, the transposable elements, and the annotated prophages as well as identified phagic elements in [59] are all used to calculate genome coverage statistics (see Table 2).

### Tuning the Parameters

We tuned the parameters used in the different steps based on comparisons between the bacteria Shigella flexneri 2457t serotype 2a (S. flexneri 2457t) [62] and S. typhi ty2, which are among the most difficult and the least stable genomes in the enterobacteria family, since they contain more ISs, prophages, and rearrangements than other *Escherichia* or *Salmonella* [63]. A summary of the parameters and their default values is given in Table 1. As a guideline, we took advantage of the initial coarse mapping induced by the KISs wherever possible, to help in tuning the different parameter values as described below. In addition, we ran the algorithms with a range of values and compared their behavior under the different settings.

To calibrate the parameters *gapJoinLen* and *gapExtractLen* in Step 3 of the preprocessing phase, we checked the gap distribution resulting from alignments between common unique KOs. We found that most of the gaps in these regions are usually shorter than 200 bp, with a few extending to 300 bp and 600 bp in S. flexneri 2457t and S. typhi ty2, respectively. Similarly, we checked the lengths of intragap regions in the KO alignments and found that most of these regions are shorter than 100 bp.

**Figure 7:**
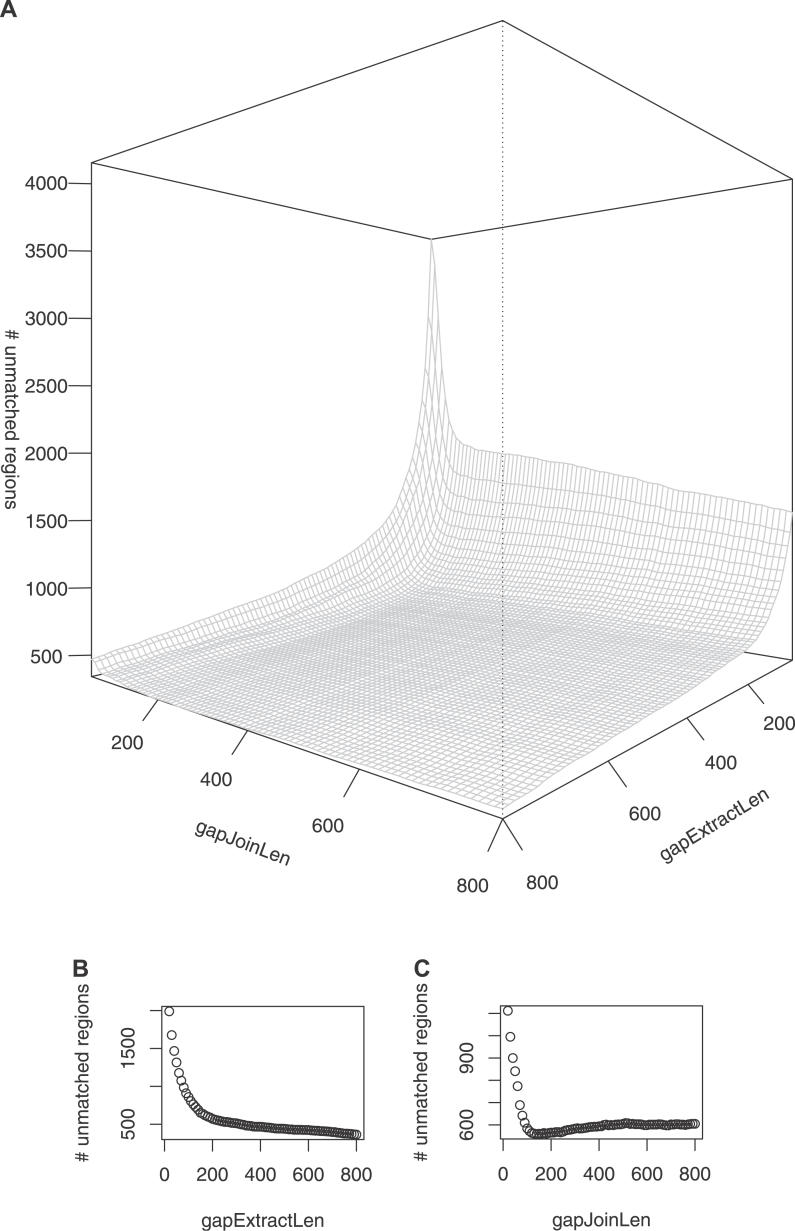
Number of Detected Unmatched Regions in Step 3 of the Preprocessing Phase (see Figure 3A and Preprocessing Phase: Building a Comprehensive Table of Similar Segments) in the Comparison of S. flexneri 2457t and S. typhi ty2 as a Function of *gapJoinLen* and of *gapExtractLen* (A) A 3-D graph of the function. (B) A projection of the graph as a function of *gapExtractLen* (horizontal axis) when setting *gapJoinLen* = 110. (C) A projection of the graph as a function of *gapJoinLen* (horizontal axis) when setting *gapExtractLen* = 200.

To gain more evidence, we ran the algorithm described in Step 3 of the preprocessing phase with different parameter values, while focusing on the total count of unmatched regions that the algorithm finds in both organisms. The results given in Figure 7 show that the count decreases rapidly as the value of *gapExtractLen* approaches 200 from below. The decrease, however, becomes moderate for values greater than 200 (Figure 7). As for *gapJoinLen,* the fast decay occurs when its value approaches 100 from below and is followed by a moderate increase for values between 200 and 600 (Figure 7). Intuitively, this change in behavior results from two factors: first, the tendency of gaps to be joined together as the *gapJoinLen* parameter increases causes the initial decrease. Second, after some point, especially when *gapJoinLen* gets larger than *gapExtractLen,* new unmatched regions longer than *gapExtractLen* start to emerge as the result of joining faraway small gaps that did not pass the threshold test before. Thus, it does not make much sense to set *gapJoinLen* to a value greater than *gapExtractLen*. In the actual runs, we used a value of 200 for *gapExtractLen* (approximately where the moderate decrease starts) and 110 for *gapJoinLen* (approximately where the minimum happens)—see Figure 7.

**Figure 8:**
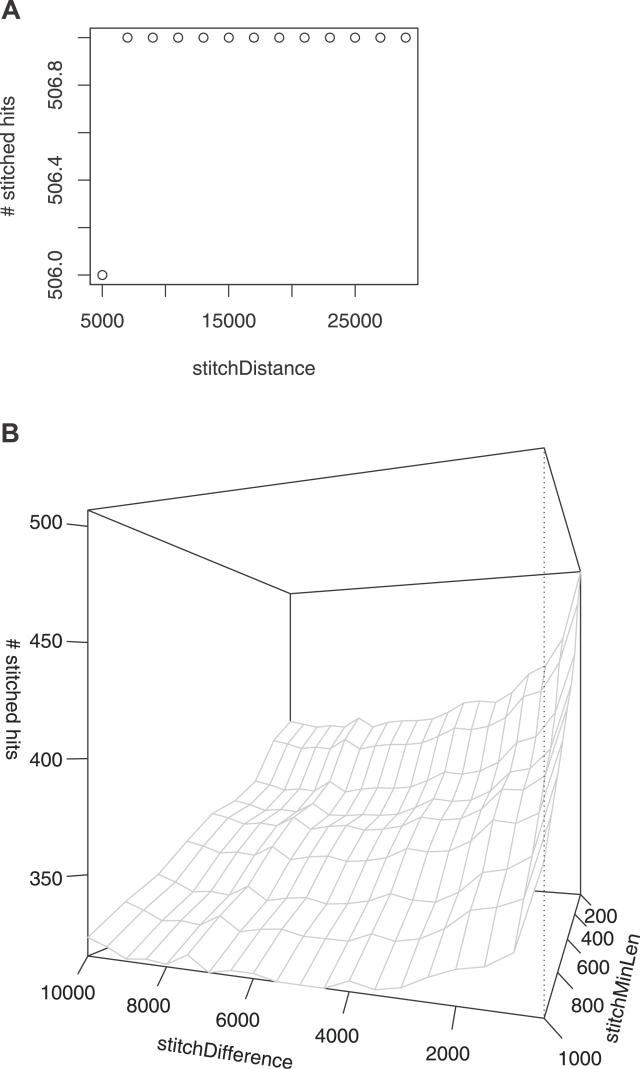
Number of Stitched Hits as a Function of the Parameters *stitchDifference, stitchDistance,* and *stitchMinLen* The hits were obtained by locally aligning inter-KIS genomic regions against the other genome in the comparison of the bacteria S. flexneri 2457t versus S. typhi ty2. (A) The number of stitched hits as a function of *stitchDistance* when *stitchDifference* = 500 and *stitchMinLen* = 200. Notice that the number of stitched hits changes only by 1 while *stitchDistance* ranges from 5,000 to 29,000. This indicates that the value of *stitchDistance* has a very weak effect on the number of stitched hits. (B) The number of stitched hits as a function of *stitchDifference* and *stitchMinLen* when *stitchDistance* = 29,000. Here, the number of stitched hits decreases rapidly as *stitchDifference* increases from 1,000 to 2,000. This is followed by a moderate decrease. As *stitchDifference* approaches 8,000, a moderate increase is observed. The reasons for this increase are similar to those discussed in Tuning the Parameters to explain the increase observed in Figure 7. On the other hand, and as is expected, the number of stitched hits decreases monotonically when *stitchMinLen* runs from 200 to 1,000.

Similar runs were performed to determine the value of the parameters *stitchDifference, stitchDistance,* and *stitchMinLen* used in Step 4 of the preprocessing phase. The results, shown in Figure 8, showed that the parameter *stitchDistance* did not have much influence on the number of stitched hits calculated by the algorithm. In the actual runs, we used values of 2,000, 200, and 15,000 for the parameters *stitchDifference, stitchMinLen,* and *stitchDistance,* respectively. Loose values can be chosen for these parameters since the stitched hits are going to be validated by global alignments.

**Figure 9:**
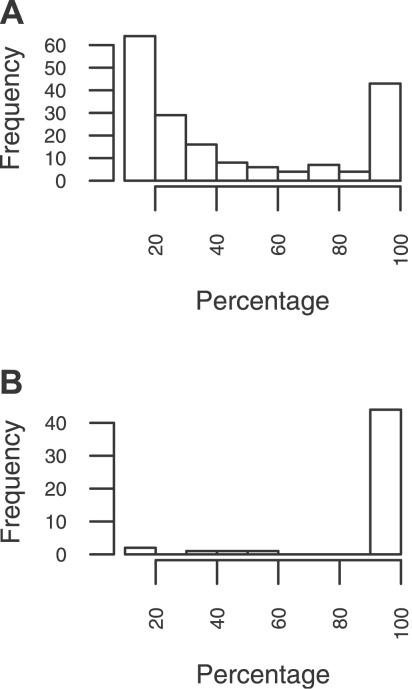
Step 1 of the Mapping Phase in the Comparison of S. flexneri 2457t and S. typhi ty2 Histograms of the percentage of intersection (horizontal axis) between entries of the comprehensive table and transposable elements (A) and between entries of the comprehensive table and phages (B). See Figure 3 and Step 1: Removing trivial entries and selfish DNA.

**Figure 10:**
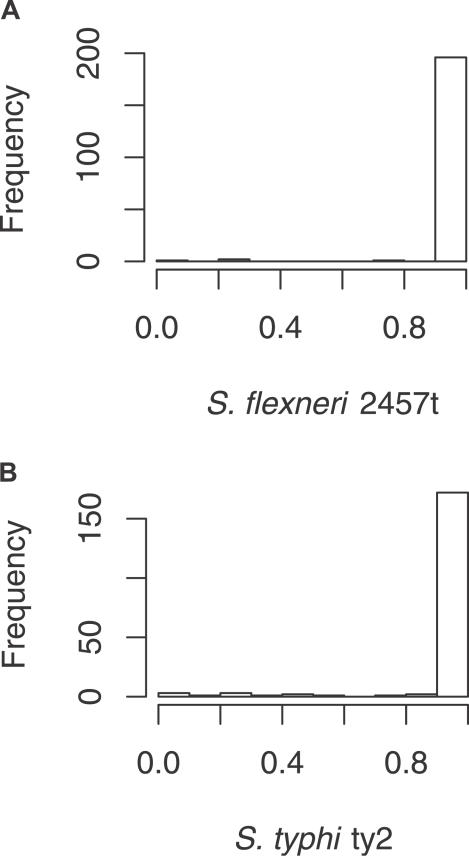
Histogram of Intersection Ratios between Table Entries (after Removing Trivial Entries and Selfish DNA) in the comparison of S. flexneri 2457t and S. typhi ty2 The ratio is calculated as the intersection length divided by the length of the shortest entry. (A) Ratios in S. flexneri 2457t and (B) ratios in S. typhi ty2. We set the default value of *dupPerc* to 50% since most of the histogram values are larger than that.

**Figure 11:**
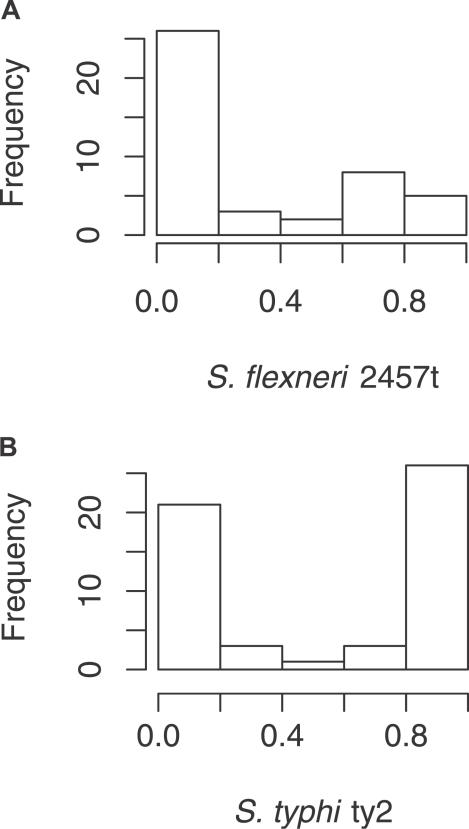
Histogram of Length Ratios between Significant Overlaps (after Removing Nuisance Cross-Overlaps) in the Comparison of S. flexneri 2457t and S. typhi ty2 Given two entries that significantly overlap, the ratio between the smallest entry to the largest one is calculated. For identifying significant overlaps we use *dupPerc* = 50%. (A) Ratios in S. flexneri 2457t and (B) ratios in S. typhi ty2. We set the default value of *orthPerc* to 50%, where the minimum in both histograms occurs.

The value of *cleanMinLen* was set to equal that of *stitchMinLen* (200 bp). Tuning the values used for discarding transposable elements and prophages was based on the intersection percentage between table entries and identified transposable elements as well as that between table entries and annotated prophages, respectively. Histograms describing these percentages are given in Figure 9. As mentioned earlier, these elements' sequences diverge fast. Therefore, one would expect that they undergo rapid changes in their sequence. To balance this effect, we choose a conservative threshold for their identification, by setting both *cleanISPerc* and *cleanProPerc* to 40%. The values of *dupPerc* and *orthPerc* were both determined to be 50%, based on similar histograms (see Figures 10 and 11).

For identifying local similarities between extracted unmatched regions and whole genomes, we used blast [54] in its bl2seq [64] implementation, with an e-value of .01.

### A Comparison with Mauve's Procedure

We compare MAGIC primarily with Mauve, a cross-species genome comparison tool, since the motivation for developing Mauve was to compare bacterial genomes. In fact, Mauve was initially used on nine enterobacterial genomes, four of which form two pairs that are considered in this study. In addition, it has been previously shown that Mauve is more accurate than other well-known comparison tools on bacteria pairs with substitution and inversion rates as in the above nine enterobacteria genomes [14].

#### Preprocessing phases.

In the preprocessing phase, Mauve [14] constructs multiple maximal *unique* matches (multi-MUMs) and considers only those that are longer than a given threshold as alignment anchors. The multi-MUMs correspond to *exact* matches shared by a number of genomes. Mauve uses a recursive anchoring scheme combined with its mapping phase. One can pinpoint at least two differences between the preprocessing phase of Mauve and our method: first, MAGIC does not require exact matches; this allows comparing more divergent genomes [14]. Second, MAGIC does not consider only unique matches. On the contrary, it aims towards building a comprehensive table.

#### Mapping phases.

Mauve applies a selection criterion to the multi-MUMs calculated in the preprocessing phase (see the previous section Preprocessing phases). Before that, overlaps between the multi-MUMs are resolved *locally,* i.e., by only considering the two overlapping multi-MUMs. Then, a minimum partitioning of the multi-MUMs into collinear blocks (i.e., segments free of reordering) is done by breakpoint analysis [65]. To do that, a guiding phylogeny is calculated from the multi-MUMs. After calculating the partitioning, the locally collinear blocks (LCBs) having the minimum weight are discarded if their weight is below a given threshold. The weight of an LCB is defined to be the sum of lengths of the multi-MUMs contained in it. After discarding the minimum weight blocks (if any), the program recalculates the partitioning and discards the minimum weight blocks iteratively. Thus, Mauve's mapping phase requires that the input table is one-to-one, which is guaranteed by locally resolving overlaps. Our approach resolves overlaps based on global information, after performing a clustering step. In contrast to our approach, Mauve tackles the problem of selfish DNA, similarly to GRIMM-Synteny and CHAIN-NET, by discarding blocks weighing less than a given threshold. Similarly to our method, in Mauve gaps are extracted from the alignments to construct the “backbone” of the compared genomes. However, in Mauve proximal gaps are not grouped prior to their extraction.

There is at least one important difference between the mapping phases of Mauve and MAGIC: in MAGIC, resolving overlaps is done based on global information. Thus, given two overlapping entries, MAGIC seeks evidence on how to solve the overlap in other table entries. Mauve, on the other hand, resolves the overlap based on local information and by considering only the two overlapping entries. The section A Comparison with Mauve's Results demonstrates the implications of this difference.

### Example Run

To illustrate MAGIC's operation and output we use the same pair, i.e*.*, S. flexneri 2457t and S. typhi ty2, that was used in Tuning the Parameters. Yet, and for the same reason, its results are excluded from the subsequent biological discussions (to avoid tuning the algorithm and analyzing its results on the same input).

The genome of S. flexneri 2457t contains 4,599,354 bp, 4,068 genes, and 131 RNAs. Out of the 4,068 genes, 1,446 are associated with KOs. The genome of S. typhi ty2 is somewhat larger: it contains 4,791,961 bp, 4,323 genes, out of which 1,542 are associated with KOs, and 109 RNAs. The number of common KOs is 1,118, out of which 917 are unique. The KIS permutation contains 69 runs and covers about 77% of both genomes. The total length of the homologous segments in these KISs, i.e., the sum of the lengths of the 917 common KOs covers slightly more than 20% of the two genomes. For comparison, see Table 5, which gives a complete overview on genome coverage statistics reported by MAGIC.

**Table 5:**
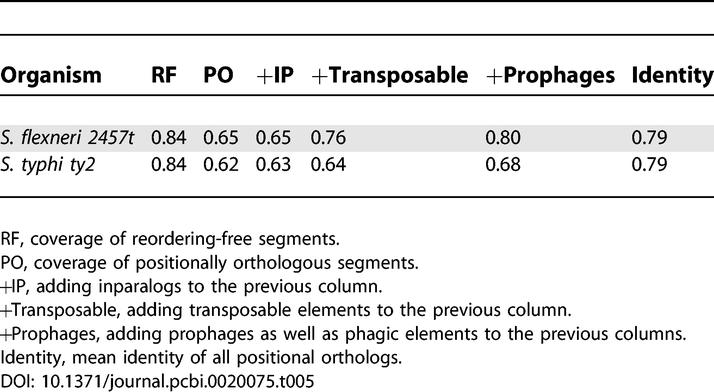
Genomes' Coverage Statistics

After running the preprocessing phase and removing short entries, the comprehensive table of similar segments contained 854 entries. The number of entries identified as belonging to transposable elements was 72. The number of entries identified as belonging to prophages was 45. Entries belonging to either of the two categories were discarded from the table leaving 737 entries. Notice that these numbers are *not* the number of transposable elements or prophages in either of the two genomes: transposable elements or prophages that are present in one organism but not in the other are not expected to be included in the mapping. Furthermore, as mentioned earlier, two segments belonging to the same prophage in one organism might correspond to two distant—either chromosomal or phagic—segments in the other organism.

Joining overlapping entries and clustering consecutive entries reduced the table size to 247. After removing identified nuisance cross-overlaps, the size is further reduced to 185. Classifying the inparalogs resulted in 22 and 19 identified duplications in the two bacteria, respectively. Some of the duplicates are rather nontrivial. A detailed description of the nontrivial duplicates is given in Text S1. We note that the table converged with respect to nuisances and inparalogs after the first iteration (see Figure 3 and Steps 3–5: Identifying nuisance, classifying inparalogs, and re-clustering).

**Figure 12:**
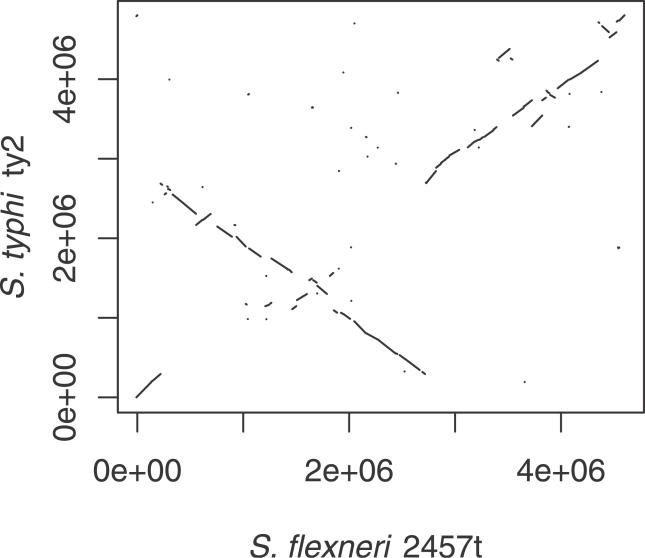
A Dot-Plot-Like Schematic Presentation of the Final One-to-One RF Table in the Comparison between S. flexneri 2457t (Horizontal Axis) and S. typhi ty2 (Vertical Axis) Each line in the figure corresponds to an entry in the table. Lines corresponding to entries with a positive sign are drawn parallel to the positive diagonal and lines corresponding to entries with a negative sign are drawn parallel to the negative diagonal.

The table size after removing the classified inparalogs is 140. After clustering consecutive entries a second time, the table size is reduced to 122. The number of remaining unresolved significant overlaps is nine. The total lengths of these entries are 5,920 bp (.1%) and 3,962 bp (.08%) in the two bacteria, respectively. They are discarded, and clustering consecutive entries is done for the last time, which results in the final table of size 106. A schematic presentation of the comparative mapping is given in Figure 12.

Recall that there were 201 entries in the initial anchor set corresponding to common non-unique KOs. Except perhaps for some of the identified duplications and some of the nine unresolved significant overlaps, MAGIC was able to establish that the ambiguity in the majority of the non-unique common KOs does not result from inparalogs. Thus, it most likely results from nuisance cross-overlaps or selfish DNA elements.

The calculated statistics of the two genomes is given in Table 5. The RF coverage leaves about 16% of both genomes in the breakpoint regions. A comparison between the RF coverage and the positional ortholog coverage shows that a significant amount of indels (about 20%) is found inside RF segments. Notice that taking the inparalogs into account did not add much to the positional ortholog coverage percentage. On the other hand, transposable elements do cover a significant part of S. flexneri 2457t—about 10%—as previously indicated [62]. However, they are less significant in S. typhi ty2. Prophages contribute equally to the coverage of both genomes—about 5%. The average nucleotide similarity between positional orthologs in both genomes equals 79%. Notice that approximately 20% and 30% of the genomes of S. flexneri 2457t and S. typhi ty2, respectively, can neither be mapped onto the other genome nor explained by known transposable elements or prophages. These portions can result from deletions, unknown prophages, unknown transposable elements, or HGTs. Interestingly, several authors [66,67] estimate that E. coli (which is the same species as S. flexneri) has obtained >15% of its genetic material by HGT from distant species having different sequence composition.

### False Anchors

Even when MAGIC starts from a high quality anchor set (the KOs) and the organisms are evolutionarily close, a few false anchors could still be discovered. A striking example was found in the comparison of Salmonella typhi CT18 (S. typhi) and S. typhi ty2 (99% average identity)—see Figure S2. K00357 (hmp), dihydropteridine reductase, one of the anchors uniquely common to the two organisms, corresponds to genomic segments (621146,621799) and (344300,345490), respectively. This anchor is isolated (it is not collinear to any other anchor). Furthermore, the identity percentage achieved by globally aligning the two segments is about 56% and 31%, respectively. Notice that in KEGG [38], unlike in MAGIC, the alignments are done on the translated amino acid sequences, which may result in high amino acid similarity for low DNA sequence similarity. MAGIC, however, has found the segment (595530,629581) in S. typhi to correspond to (2351520,2385571) in S. typhi ty2. These two segments are 99% identical, are much longer than the initial KO, and are collinear with other entries. As for the segment (344300,345490) in S. typhi ty2, MAGIC has found the segment (344119,355499) in S. typhi ty2 to correspond to (2649350,2660730) in *S. typhi,* with similar properties to the above. Thus, K00357 is an example of a false anchor and a nuisance cross-overlap. As such, it is discarded during MAGIC's mapping phase (see Steps 3–5: Identifying nuisance, classifying inparalgos, and re-clustering).

Detailed statistics and classifications of false anchors are given in A Comparison with Mauve's Results and in Table 4.

### Major Forces Shaping the Prokaryotic Genome

The results described in Table 2 enable us to compare between the different forces shaping the genome, i.e*.*, point mutations, genome reorderings, duplications, and indels (insertions resulting from transposable elements, prophages, and HGTs or deletions). Many of these elements have been separately analyzed in the recent literature [55,58,59,68], but were not quantitatively compared against each other.

A comparison between duplications and indels indicates that indels are more frequent. Duplications, except in Bordetella bronchiseptica and the two *Neisseria,* account for at most 1% of the genomes' lengths, whereas a large fraction of the genomes are not mapped to their counterparts because of lack of similarity. This observation is best explained by HGT or deletion events and is coherent with data indicating that HGT is the major cause of bacterial diversification [69]. It is also consistent with the known role of lateral transfer in the fast exchange of pathogenicity islands among prokaryotes [70,71] and supports the hypothesis regarding its central role in speciation and sub-speciation [72–75]. In *B. bronchiseptica,* these events account for up to 30% of its genome. This high percentage is likely to result from massive gene loss in Bordetella pertussis due to the action of transposable elements and its small population size [76]. This analysis also points out that gene loss in B. pertussis has severely reduced the number of inparalogs relative to the ancestral genome, if this is taken to be more similar to B. bronchiseptica. Taking into account the evolutionary distance between the pairs of genomes, one finds *Buchnera* (B. aphidicola aps and B. aphidicola sg) at the other end of the spectrum with as little as 7% of unmapped regions for an average sequence identity of 75%, as described previously [77].

Transposable elements and prophages can account for as much as 18% of the genome length, as in S. pyogenes ssi1. However, the share of prophages is typically much larger than that of transposable elements, namely 3% and 1.25% on average, respectively.

### Breakpoint Distribution

Let *x*_1_ < *x*_2_ < . . . < *x_k_* be the ordered positions of *k* breakpoints relative to a fixed origin in the genome, and denote the genome length by *G*. The check for breakpoint circular uniformity was done on the transformed positions 2*πx*_1_/*G*, 2*πx*_2_/*G*, …, 2*πx_k_*/G by applying the Rao, the Watson, and the Kuiper tests [78,79]. The critical value in the three tests is set to 5%. All tests showed similar tendencies, except on the *Helicobacter* pair, S. flexneri 2457t, and Neisseria meningitidis a (which Rao rejected while Watson and Kuiper accepted), demonstrating the robustness of the results. In Table 2 we report the Rao test results (as mentioned above, the Rao test rejected more organisms than the other two tests), and the discussion is based on them. These results indicate that in more than four pairs, three of which are considered reliable (see Table 2), one cannot reject the uniformity null-hypothesis.

One should emphasize that the breakpoint uniformity test aims only at checking genome reorderings. It does not take into account the disruption of chromosomal organization resulting from deletions and insertions of genetic material. In our comparisons we find that between 3% and 30% of the genomes cannot be mapped either to their counterpart or to known selfish DNA (Table 2). These regions are likely to correspond to indels that occurred since the divergence of the species, i.e., HGTs and large deletions, and they have an important role in defining the boundaries between RF segments. Hence, our observation that the distribution of breakpoints can be uniform suggests that, in the relevant cases, the distribution of reorderings, and also the distribution of HGTs and large deletions, is uniform. This is in line with data indicating that HGT is more or less homogeneously distributed in E. coli [80] and along the two replichores of *Bacillus* and *Streptococcus* [81].

Interestingly, and despite containing noninvertible segments [20–23], both S. typhimurium and E. coli mg1655 passed the uniformity test. The fit of uniformity in four pairwise comparisons is surprising for two additional reasons. First, one does not expect deletion and reordering events to be totally random because both events are typically induced by homologous recombination between repeated elements [27]. Since these are discrete elements dispersed along the genome, the likelihood of each pair of contiguous nucleotides in the genome to be separated by a reordering mutation is not homogeneous. However, if there are many pairs of repeats displaced randomly in the chromosome, each with a low probability of leading to a reordering mutation, the overall picture is one of random distribution of breakpoints and few—if any—breakpoint reuses. Indeed, both E. coli mg1655 and S. typhimurium have many repeats [82], but the number of reorderings between them is very low, in spite of the ~100 million years of divergence [83]. Hence, although repeats are abundant and lead to reorderings, each individual repeat has a low probability of leading to a reordering that gets fixed in the population [84]. Also, when one identifies inverted repeats within a genome, the distance between each pair of repeats is random [82]. Thus, the conditions for a uniform positioning of breakpoints are fulfilled, but at a coarser level—the one defined by the distribution of repeats in genomes.

Second, even if reorderings, HGTs, and deletions were random, one would expect selection to purge events disrupting operons [85] or disorganizing the chromosome relative to replication [81]. However, none of these constraints would lead to a large deviation from uniformity. Hence, our data suggest that the uniformity is a good approximation of the reordering mechanisms of closely related bacterial genomes, even though a finer analysis might reveal the selective constraints associated with the organization of the genome.

### A Comparison with Mauve's Results

Recall that Mauve's output includes an alignment of LCBs, comparable to MAGIC's RFs, and a backbone table (comparable to MAGIC's positional orthologs) containing segments in these alignments that do not contain large gaps. These segments are extracted similarly to Step 3 of MAGIC's preprocessing phase. However, in Mauve, proximal gaps are not joined before extracting long ones *(>maxBackboneGapSize*). In addition, similarly to MAGIC's filtering steps, Mauve discards short entries *(<minBackboneSize*). The seed-size threshold used in Mauve (comparable to BLAST's seed size) for filtering short multi-MUMs is dynamically calculated by the formula *seed_size =* log_2_ 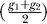 /1.5, where *g*_1_ and *g*_2_ are the sizes of the first and second genomes, respectively. For the genomes considered in this study, this value ranges between 12.8 and 15. These three parameters are comparable to the three parameters *gapExtractLen, stitchMinLen,* and BLAST seed size used in MAGIC, respectively. By giving these parameters two sets of values, we conducted two runs: in the first, Mauve's default values are used, whereas in the second, we set the values of the parameters to the defaults used in MAGIC (*seed_size* = 11, *maxBackboneGapSize* = 200,*minBackboneSize* = 200). The results of both runs are given in Tables S1 and S2. We note that these settings might not be optimal for Mauve and that other settings might yield better results. A comparison between the results of both runs of Mauve (Tables S1 and S2) against MAGIC's results (Table 2 and the example run given above in Example Run) shows the following: a) Mauve fragments the genomes into more segments (~7.7-fold higher on average in the ten pairs); b) Mauve's default settings sometimes give poor coverage—see, for example, the *Streptococcus* pair (with LCB/backbone coverage of 43% and 39%, respectively, in the default run and 95% and 92%, respectively, in the nondefault run); c) changing the seed size usually increases the fragmentation of the genomes as well (e.g., the number of LCBs is 9-fold higher for the *Buchnera* pair); d) both runs of Mauve reject the breakpoint circular uniformity null-hypothesis in all pairs, except in *Buchnera* (which has a single RF according to MAGIC); e) the values of the identity percentage in the three tables (Tables 2, S1, and S2) are similar; f) the LCB and backbone coverage of Mauve with nondefault parameters are similar to the RF and positional orthologous coverage of MAGIC, respectively; g) Mauve is faster than MAGIC (unpublished data). On the example run (the comparison of S. flexneri 2457t with S. typhi ty2), Mauve terminated after less than 17 min of CPU time (the run was done by using the command-line tool, called mauveAligner—and not the Java GUI, called Mauve—as well as by using the program calculateBackboneCoverage for extracting the backbones and calculating identities), while MAGIC required about 35 min of CPU time, most of which is consumed by AVID for global alignments (about 70%) and by BLAST for local alignments (about 13%). Recall that the rest of MAGIC's steps are implemented as R [39] and BASH scripts, whereas Mauve is implemented in C. (We expect MAGIC's C++ implementation to be faster.)

The differences in the LCB/RF numbers may result from either of these two reasons: the methods cover rather different parts of the genomes or the methods cover similar parts but map them differently. To get a better understanding of the differences in this case, we conducted two types of analysis: first, we checked whether the genomic portions covered by the two methods are similar. Second, we checked whether the mappings are similar. The results of the first comparison are given in Table 3 for Mauve's default run, and in Table S3 for Mauve's nondefault run. These two tables show that when the coverage percentage is similar (e.g*.*, not in the *Streptococcus* pair), both methods cover similar portions of the genomes. Thus, the difference between the methods is most likely in the mapping.

To gain more evidence for this, we utilized the flexibility of MAGIC and its ability to correct false anchors by running it on Mauve's backbone tables. This feature is made possible by MAGIC's robustness with respect to the initial set of anchors, which we discuss in the next section. By checking MAGIC's classifications of the backbone anchors, we are able to learn more about the differences in the mappings. Table 4 describes these classifications. The table shows that there are indeed differences between the mappings achieved by the two methods. These differences are significant enough to explain the deviations in the results of the uniformity test. For example, in genomes with abundant transposable elements, e.g., *Shigella,* the results show many of Mauve's backbone anchors corresponding to transposable elements. A similar situation exists in genomes abundant with prophages, e.g., *Streptococcus*. In genomes abundant with repeats, e.g*.*, *Neisseria* [86], the results show many of Mauve's backbone anchors corresponding to nuisance cross-overlaps, inparalogs, or final conflicts that MAGIC could not resolve (see Steps 6–7: Discarding remaining conflicts and re-clustering).

We note that for the two enterobacteria pairs, the total percentage of the corrected anchor lengths is less than 4%. This percentage is in agreement with the estimated error for Mauve on the enterobacteria family [14]. The results achieved by both methods show that this small percentage (calculated according to nucleotide lengths) causes considerable differences in breakpoint positions, which are very significant for the uniformity test.

### MAGIC's Robustness

MAGIC robustness was checked against two types of changes: first, and since MAGIC is an anchor-based method, robustness with respect to the initial set of anchors was tested; and second, robustness with respect to perturbations in the parameters' values was tested. In the following, we report the results of both tests.

#### Robustness with respect to the initial anchor set.

To validate the robustness of MAGIC relative to the initial set of anchors, we ran it on the backbone tables produced by Mauve (with default parameters). The results of this run are given in Table S4, and are similar to the results achieved in the KO-based run (Table 2). This similarity demonstrated the robustness of MAGIC: in both runs, the identities are equal. The difference in RF numbers is at most 11 (in the *Pyrococcus* archaeal pair where the RF number equals 88 and 99, respectively). The difference in the RF coverage is usually very low and reaches a maximum of 5% in Neisseria meningitidis b. The positional ortholog coverage is usually very similar and its maximal difference (of 7%) is obtained in S. pyogenes m18 (which belongs to the pair on which Mauve yielded very low coverage with the default settings—see Table S1). Finally, the paralog percentage (the difference between the +IP column and the PO column in Table S4) is virtually identical.

The test for uniformity yielded similar results, and the same pairs are accepting/rejecting the test in both MAGIC's runs.

#### Robustness with respect to parameter values.

**Figure 13:**
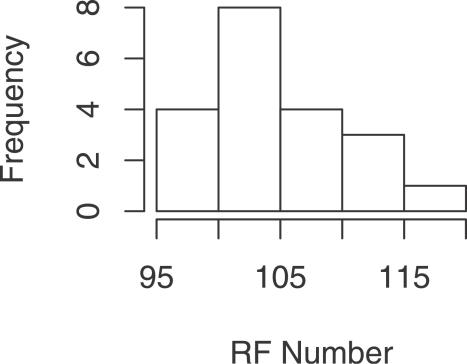
Histogram of RF Numbers when Comparing S. flexneri 2457t versus S. typhi ty2 under Different Parameter Values See the section Robustness with respect to parameter values.

We checked the effect of changing the parameters' values on MAGIC's results. Each length parameter was randomly chosen from {1/2*d*,*d*,3/2*d*}, where *d* is the parameter's default value (Table 1). For example, *gapJoinLen* was randomly chosen from {55*bp*,110*bp*,165*bp*}. Each percentage parameter was randomly chosen from {*d* − 0.1,*d*,*d* + 0.1}, where *d* is the parameter's default value. For example, cleanISPerc was randomly chosen from {0.3,0.4,0.5}. Twenty random runs were performed on S. flexneri 2457t and S. typhi ty2. The greatest deviations were observed in the number of RFs—see Figure 13. These changes are not surprising, and result from removing or adding different types of entries along MAGIC's run because of the different parameter values. On the other hand, the RF and the positional ortholog coverage did not change significantly, ranging 0.83–0.85 and 0.59–0.64 in S. flexneri 2457t, and 0.83–0.85 and 0.57–0.62 in S. typhi ty2, respectively. The identity did not change much either, and ranged 0.78–0.80 in S. flexneri 2457t and 0.78–0.79 in S. typhi ty2.

### MAGIC's Scalability

We perform two tests to check how MAGIC scales up: first, we run MAGIC on evolutionary distant pairs. Second, we test MAGIC on large genomes.

#### Comparing evolutionarily distant organisms.

The results of comparing evolutionarily distant organisms are given in Table 6. Despite their very low average similarity (~65% of protein similarity between orthologs), MAGIC was able to calculate a good mapping for these pairs. In particular, the results for the *Chlamydia* pair are in agreement with those reported in previous studies [87].

**Table 6:**
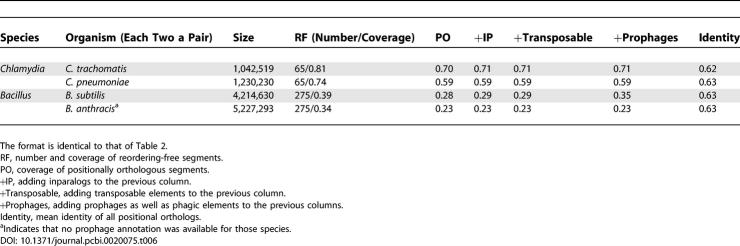
Comparing Distantly Related Bacteria Pairs

#### Comparison of the X chromosome in human–mouse.

Although the initial motivation of our method was to compare prokaryotic genomes, we applied MAGIC to the X chromosome of the human and mouse genomes to assess its scalability. Running the preprocessing phase on the human–mouse genomes is time-consuming: BLASTZ (a fast local alignment tool) required 481 days of CPU time on a cluster of 1,024 833-Mhz Pentium III CPUs [12]. To avoid this computational bottleneck, we extracted the information on the X chromosome from the precomputed preprocessing table available on the UCSC Genome Browser [88] for the human genome build 35 and the mouse genome build 35. Because of the large size of the table, we had to implement our mapping phase in C++. The initial size of the table (restricted to the X chromosome) is 86,755. Running the C++ implementation required less than half a minute of CPU time and less than 10 MB of memory on a 1.4-GHz PC equipped with GNU/Linux. The resulting table contained 1,101 entries. Removing entries shorter than 100,00 bp and re-clustering reduced the table size to 25. Removing entries shorter than 1,000,000 bp and re-clustering reduced further the table size to 7. This table is presented in Table S5. The calculated RF coverage is 0.85 and 0.90 in the human and mouse, respectively. These results are comparable to what has been previously reported [17,88]—see Tables 7 and 8. The implementation of MAGIC in C++, when ready, will allow both a more detailed comparison of these results and an extension of the analysis to larger chromosomes.

**Table 7:**
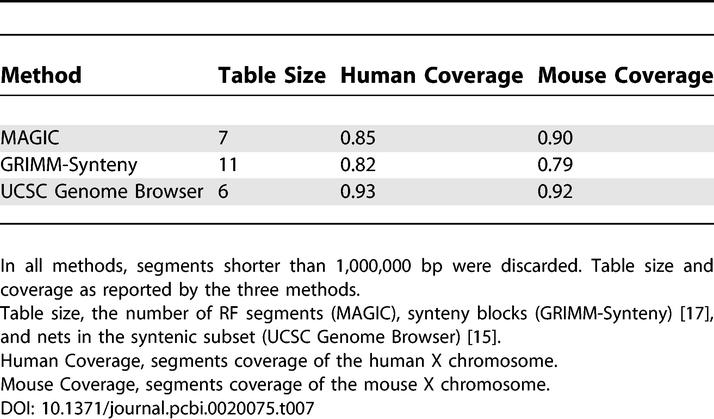
Comparing the Results of MAGIC, GRIMM-Synteny, and the UCSC Genome Browser on Human–Mouse X Chromosome

**Table 8:**
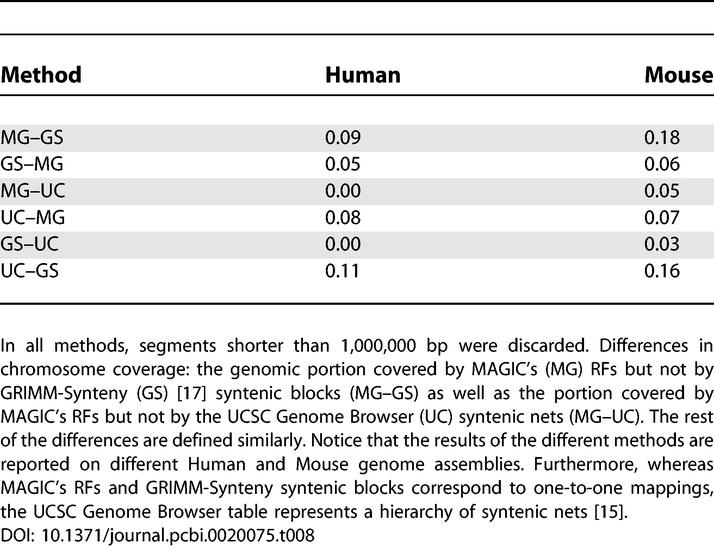
Comparing the Results of MAGIC, GRIMM-Synteny, and the UCSC Genome Browser on Human–Mouse X Chromosome

### Future Work

MAGIC can be enhanced to enable comparing both multichromosomal organisms and multiple genomes. Furthermore, MAGIC can be extended to integrate additional annotations of the genomes. For example, such an approach was adopted in [89]. Here, identified repeat pairs were added to MAGIC's comparative mapping, enabling a unique and efficient reconstruction of the ancestral genome order and the rearrangement scenario.

## Materials and Methods

In this section we give a formal description of the methods used in stitching local matches (Step 4 in the preprocessing phase) and clustering the RF segments (Step 2 in the mapping phase). Throughout the section we present a correspondence between a segment in organism 1 and a segment in organism 2 as an Entry *I* = ((*s*_1_(*I*),*e*_1_(*I*)),(*s*_2_(*I*),*e*_2_(*I*)),*sign*(*I*)). Here, (*s_k_*(*I*),*e_k_*(*I*)) represents the start and end indices of the entry in organism *k,* for *k* ∈ {1,2}, and *sign*(*I*) represents the sign of the entry, i.e., +1, if the matching sequences have the same orientation in both organisms and −1 otherwise.

### Formal description of stitching local matches.

Let *I* and *J* be two entries representing two hits such that *sign*(*I*) = *sign*(*J*), *s*_1_(*I*) ≤ *s*_1_(*J*), and *sign* (*I*) · *s*_2_(*I*) ≤ *sign*(*I*) · s_2_(*J*). We define the relative distance between (*s*_1_(*I*),*s*_1_(*J*)) and (*s*_2_(*I*)*s*_2_(*J*)) as


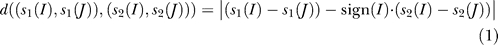


Notice that *s*_2_(*I*) ≤ *s*_2_(*J*) when *sign*(*I*) = 1. But if *sign*(*I*) = −1, we have *s*_2_(*I*) ≥ *s*_2_(*J*). To correct the latter, we need to multiply *s*_2_(*I*) − *s*_2_(*J*) by the sign *sign*(*I*).

Intuitively, the distance defined in Equation 1 corresponds to the length of indel mutations that occurred since the divergence of the two organisms. This distance directly influences the result of the global alignment that we are going to perform on the stitched local matches. If the distance in Equation 1 is too big, then most probably the global alignment is going to contain big gaps, and hence is going to be broken. Thus, stitching faraway (with respect to Equation 1) hits would be unwise. Therefore, we first group hits with relative distance smaller than a specified threshold (parameter *stitchDifference* in Algorithm 2). Afterward, we check that the grouped hits are not too far apart on both genomes, i.e., that | *s*_1_(*I*) − *s*_1_(*J*)| + | *s*_2_(*I*) − *s*_2_(*J*) | is smaller than a specified threshold (parameter *stitchDistance* in Algorithm 2). Indeed, if a segment is conserved in the two organisms, one would expect to find a lot of hits in it. Thus, the distance between consecutive hits in a conserved segment should be short. Finally, stitched matches that are longer than a specified threshold in both organisms (parameter *stitchMinLen* in Algorithm 2) are further considered. Pseudo-code describing this step is given in Algorithm 2.

### Formal description of clustering into RF segments.

Figure 2 is used throughout this section to demonstrate the different definitions and steps.

Denote the length of the intersection of Entries *I* and *J* in organism *k* by *intersect*(*I*,*J*,*k*) and denote the length of Entry *I* in organism *k* by λ(*I*,*k*). Entries *I* and *J* are said to *significantly overlap* in organism *k* if their intersection length is greater than *dupPerc* of the length of the shorter one according to *k,* i.e., *intersec*t(*I*,*J*,*k*) ≥ *dupPerc* · min(λ(*I*,*k*),λ(*J*,*k*)). Define *Duplicates*(*I*,*k*) to be the set of all entries that significantly overlap with Entry *I* in organism *k* (including *I* itself). In Figure 2 (with *dupPerc* = 50%), since Entries 2 and 3 significantly overlap in *E. coli,* we have *Duplicates*(2,1) = {2,3}. In S. typhi ty2, however, Entry 2 does not overlap significantly with other entries and thus *Duplicates*(2,2) = {2}. Given two Entries *I* and *J,* denote by *dist*(*I*,*J*,*k*), for *k* ∈ {1,2}, the offset between *J* and *I* when sorting the table in lexicographical order according to (*s*_k_,*e*_k_), i.e., sorting first according to *s_k_* and among entries having the same *s_k_* value according to *e_k_*. For example, if the table is sorted according to (*s*_1_,*e*_1_), then *dist*(*I*,*J*,1) = J − *I*. Notice that dist(·,·,·) is a signed quantity. In Figure 2, the arrows are sorted in lexicographical order according to E. coli. For Entries 1,2 and 4 we have: *dist*(1,2,1) = *dist*(1,2,2) = 1 while *dist*(1,4,1) = 3 and *dist*(1,4,2) = −2 (Entry 3 precedes Entry 4 in S. typhi ty2).

We refer to two Entries *I* and *J* such that | *dist*(*I*,*J*,*k*) | = 1 as *successive* in organism *k*. If *dist*(*I*,*J*,1) = 1 and *dist*(*I*,*J*,2) = sign(*I*), then *I* and *J* are successive in both organisms in the correct orientation with respect to *I*'s sign. If, in addition, *sign*(*I*) = *sign*(*J*), then (*I*,*J*) are *consecutive*. If the table does not contain overlapping entries, the previous definition would have been sufficient. However, as Figure 2 demonstrates, usually this is not the case. To cope with overlapping entries, the definition of consecutive entries needs to be generalized. We denote the “desired” distance between two Entries *I* and *J* that have the same sign by *C_k_*. So we have *C*_1_ = 1 and *C*2 = *sign*(*I*). If the segment of *J* in organism 2 was duplicated, then we might get that | *dist*(*I*,*J*,1) | > 1, as is the case with Entries 1,3 in Figure 2. Consider the sets *Duplicates*(*I*,*k*) and *Duplicates*(*J*,*k*) for *k* ∈ {1,2}. Define the distance in organism *k* between the sets of significant overlaps of *I* and significant overlaps of *J* as the closest distance to the desired distance *C_k_* realized by a pair of significant overlaps:

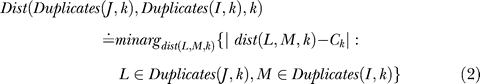
For example, we have

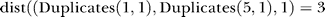
and

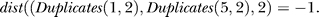



We say that (*I*,*J*) is a *potentially consecutive pair* (PCP) if J ∉ *Duplicates*(*I*,*k*) for *k* ∈ {1,2}, the entries have the same relative orientation (*sign*(*I*) = *sign*(*J*)), and


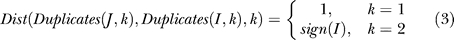


Given an Entry *I,* it might be the case that there exists *J* and *J′* ≠ *J* where both pairs (*I*,*J*) and (*I*,*J′*) are PCPs. If (*I*,*J*) is a PCP such that

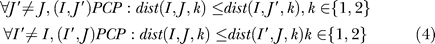
i.e., the *best* PCP, then we refer to them as *consecutive*. For a consecutive pair (*I*,*J*), where *J* comes after *I* according to the lexicographical order on (*s*_1_(·),*e*_1_(·)), we say that *J* is consecutive to *I* and denote this by *J* = *consec*(*I*).

In Figure 2, we have that *Duplicates*(1,1) = *Duplicates*(1,2) = {1} while *Duplicates*(3,1) = {2,3} and *Duplicates*(3,2) = {3,4,5}. Notice that *sign*(1) = *sign*(3) = − 1 and that Entries 1,3 do not significantly overlap. On the other hand, we have that *dist*({1},{2,3},1) = 1 and *dist*({1},{3,4,5},2) = −1. In addition (1,3) is the only PCP involving either Entry 1 or Entry 3. Thus we conclude that 3 = *consec*(1).

It is straightforward to show that, in general, if the table contains no entries that overlap in both organisms, each entry can have at most one consecutive entry and each entry can be the consecutive of at most another one.

Using the notion of “consecutive,” we can use single linkage clustering to start constructing the RF segments as follows: calculate chains (or runs) of consecutive entries in the table and join them to the same RF segment. A pseudo-code implementing this idea is given in Algorithm 3. Notice that the resulting table might still contain overlapping entries. To identify duplications correctly, we need first to identify and remove “nuisance cross-overlaps.” We say that Entry *I cross-overlaps* in the table if *I* significantly overlaps with two other Entries *H* and *J* in organisms 1 and 2, respectively, i.e., *I* ∈ *Duplicates*(*H*,1) and *I* ∈ *Duplicates*(*J*,2). Notice that cross-overlaps might correspond either to “fake” hits, e.g., low similarity homologous segments, or might be the result of evolutionary events, e.g., duplications or genome rearrangements in both organisms. We distinguish between the two cases based on the relative lengths of the entries: if the length of *I* is less than *orgPerc* the lengths of *H* and *J* in organisms 1 and 2, respectively, i.e., *ℓ*(*I*,1) < *orgPerc* · *ℓ*(*H*,1) and *ℓ*(*I*,2) < *orgPerc* · *ℓ*(*J*,2), *I* is considered as a *nuisance cross-overlap* and is discarded.

For the remaining significant overlaps, we consider those for which positional orthologs and inparalogs can be determined: if *I* significantly overlaps with *J* in organism *k* and *I* is shorter than *orgPerc* of *J*'s length in *k,* i.e., *ℓ*(I,k) < orgPerc · *ℓ*(*J*,*k*), *I* is declared an inparalog and *J* is declared a positional ortholog.

### Pseudo-code.

**Algorithm 1** getGappedSegments(*org*1*Gaps*,*org*2*Gaps*,*gapExtractLen*, *gapJoinLen*)

1: *org*1*CloseGaps* ← joinCloseGaps(*org*1*Gaps*,*gapJoinLen*)

2: *org2CloseGaps* ← joinCloseGaps(*org*2*Gaps*,*gapJoinLen*)

3: *org*1*LongGaps* ← gapsLongerThanThreshold(*org*1*CloseGaps*,*gapExtractLen*)

4: *org*2*LongGaps* ← gapsLongerThanThreshold(*org*2*CloseGaps*,*gapExtractLen*)

5: return mergeGaps(org1*LongGaps*,*org*2*LongGaps*)

**Algorithm 2** combineBlastResults(*hits*,*stitchDifference*,*stitchMinLen*,*stitchDistance*)

1: *relativeDistanceHits* ← checkRelativeDistance(*hits*,*stitchDifference*)

2: *absoluteDistanceHits* ← checkAbsoluteDistance(*relativeDistanceHits*,*stitchDistance*)

3: return checkLength(*absoluteDistanceHits*,*stitchMinLen*)

**Algorithm 3** buildRFs(*table*)

1: let *group*[·] be an array of length *length*(*table*) s.t. ∀*I* ∈ *table* : *group*[*I*]= *I*.

2: for *I* ∈ *table* do

3:  *J* ← *consec*(*I*)

4:  if J != NULL then

5:   *group*[*J*] ← *group*[*I*]

6:  end if

7: end for

8: cluster *table* according to *group*[·]

9: return clusters

## Supporting Information

Figure S1 An Example of a Final Conflict in the Comparison of S. flexneri 2457t and S. typhi ty2 The cyan and the gray entries correspond to the same segment in S. typhi ty2. However, their corresponding segments in S. flexneri 2457t are different. Since these two entries are not collinear to any other entry, one cannot infer positional ortholog and inparalog relations based on length considerations. Hence, these two entries are considered a conflict.
(30 KB PDF)

Figure S2 An Example of a False KO Anchor Resulting from a Nuisance Cross-Overlap in the Comparison of S. typhi ct18 and S. typhi ty2 Entry 2 (green) corresponds to K00357. By comparing it with Entry 1 and Entry 3, it is easy to see that Entry 2 corresponds to a false anchor and is a nuisance cross-overlap.
(21 KB PDF)

Table S1 Running Mauve with Default Parameters on 10 Prokaryotic Pairs (101 KB PDF)

Table S2 Running Mauve with Nondefault Parameters on 10 Prokaryotic Pairs (98 KB PDF)

Table S3 Comparing Coverage between MAGIC's and Mauve's Results (109 KB PDF)

Table S4 Running MAGIC while Taking Mauve's Backbone Results as Anchors (117 KB PDF)

Table S5 The One-to-One RF Table Resulting from Running MAGIC Mapping Phase and Filtering Entries of Length Smaller than 1,000,000 on the Human–Mouse X Chromosome Based on BLASTZ Chained Output as Available on the UCSC Genome Browser (37 KB PDF)

Text S1 A Description of Nontrivial Duplications in the Comparison of S. flexneri 2457t and S. typhi ty2 (40 KB PDF)

## Abbreviations

GUI: graphical user interface
HGT: horizontal gene transfer
KIS: KO-induced segment
KO: KEGG orthologs
LCB: locally colinear block
RF: reorder-free
