# Supplementary material for: An Integrative Method for Accurate Comparative Genome Mapping
Source: PLoS Comput Biol. 2006 Aug 4;2(8):e75. doi: 10.1371/journal.pcbi.0020075 (PMC1526463; doi:10.1371/journal.pcbi.0020075)
Supplement: Supplementary file 1 [file pcbi.0020075.sd001.pdf]

Text S1

In *S. flexneri* 2457t, the inparalogous segment (144889, 145107) (219bp) contained in the hypothetical *yadD* gene (or s0131), corresponds to two segments in *S. typhi* ty2: the segment (2450706, 2451000) which is contained in K05802 (AefA), an integral membrane potassium efflux system protein, and the segment (4720968, 4721186) which is contained in a putative membrane protein. Thus, these two table entries induced one “false” duplication that is discarded during the classification of the inparalogs in *S. flexneri* 2457t. Two other inparalogous segments in *S. flexneri* 2457t have a more complicated history. Segments (1413794, 1414775) (982bp) and (1985729, 1986547) (819bp), with the first partially intersecting s1450 (frameshift) and the second contained in gene *nmpC* (or s2073) coding for a putative outer membrane porin protein C precursor, both share a common similar segment in *S. typhi* ty2, that is segment (681360, 682238), which is contained in gene *ompC* (or t0597), coding for an outer membrane protein C. Moreover, the second segment has one additional similar segment in *S. typhi* ty2 which is (2605845, 2606754) contained in gene *phoE* (or t2530), coding for an outer membrane pore protein E precursor.

In *S. typhi* ty2, the inparalogous segments (2950865, 2951642) (778 bp) and (440281, 440630) (350bp) correspond to (3253352, 3254129) and a subsegment of it (3253427, 3253776) in *S. flexneri* 2457t, respectively. The first segment in *S. typhi* ty2 and the two segments in *S. flexneri* 2457t are associated with K00865 (glxK), glycerate kinase. This KO is an example of a non-unique common KO that appears in the inparalogs that were identified by our algorithm. More precisely, in *S. typhi* ty2, the inparalogous segment (2950865, 2951642) is contained in t2868, a glycerate kinase. The inparalogous segment (440281, 440630) partially intersects K01714 (dapA), a dihydrodipicolinate synthase and partially intersects NlpB (or t0371) a putative lipoprotein. In *S. flexneri* 2457t, the segment (3253352, 3254129) (and of course its subsegment) is contained in s3376 a YhaD glycerate kinase. Two other inparalogous segments in *S. typhi* ty2 correspond each to two segments in *S. flexneri* 2457t. Inparalogous segment (984225, 985099) (875bp) contained in gene *ompS* (or t0883,) coding for an outer membrane protein S1, corresponds to segments (2312562, 2313565) and (975175, 975947) in *S. flexneri* which are both associated with the KO K03285 (TC.GBP), general bacterial porin, GBP family. More precisely, the former segment is contained in gene *ompC* (or s2429), coding for an outer membrane protein 1b (Ib;c). The latter segment is contained in gene *ompF* (or s0990), coding for an outer membrane protein 1a (Ia;b;F). Finally, segment (3399221, 3401980) (2760bp) contained in gene *acrF* (or t3304) corresponds to the two segments (417659, 420585) and (2553972, 2556724) in *S. flexneri* 2457t which are both associated with the KO K03296 (TC.HAE1), hydrophobic/amphiphilic exporter-1, HAE1 family. More specifically, the former is contained in gene *acrB* (or s0414), coding for an acriflavine resistance protein. The latter is contained in gene *acrD* (or s2663), coding for a multidrug resistance protein.
