# Supplementary material for: An Integrative Method for Accurate Comparative Genome Mapping
Source: PLoS Comput Biol. 2006 Aug 4;2(8):e75. doi: 10.1371/journal.pcbi.0020075 (PMC1526463; doi:10.1371/journal.pcbi.0020075)
Supplement: Supplementary file 2 [file pcbi.0020075.sg001.pdf]

Figure S1

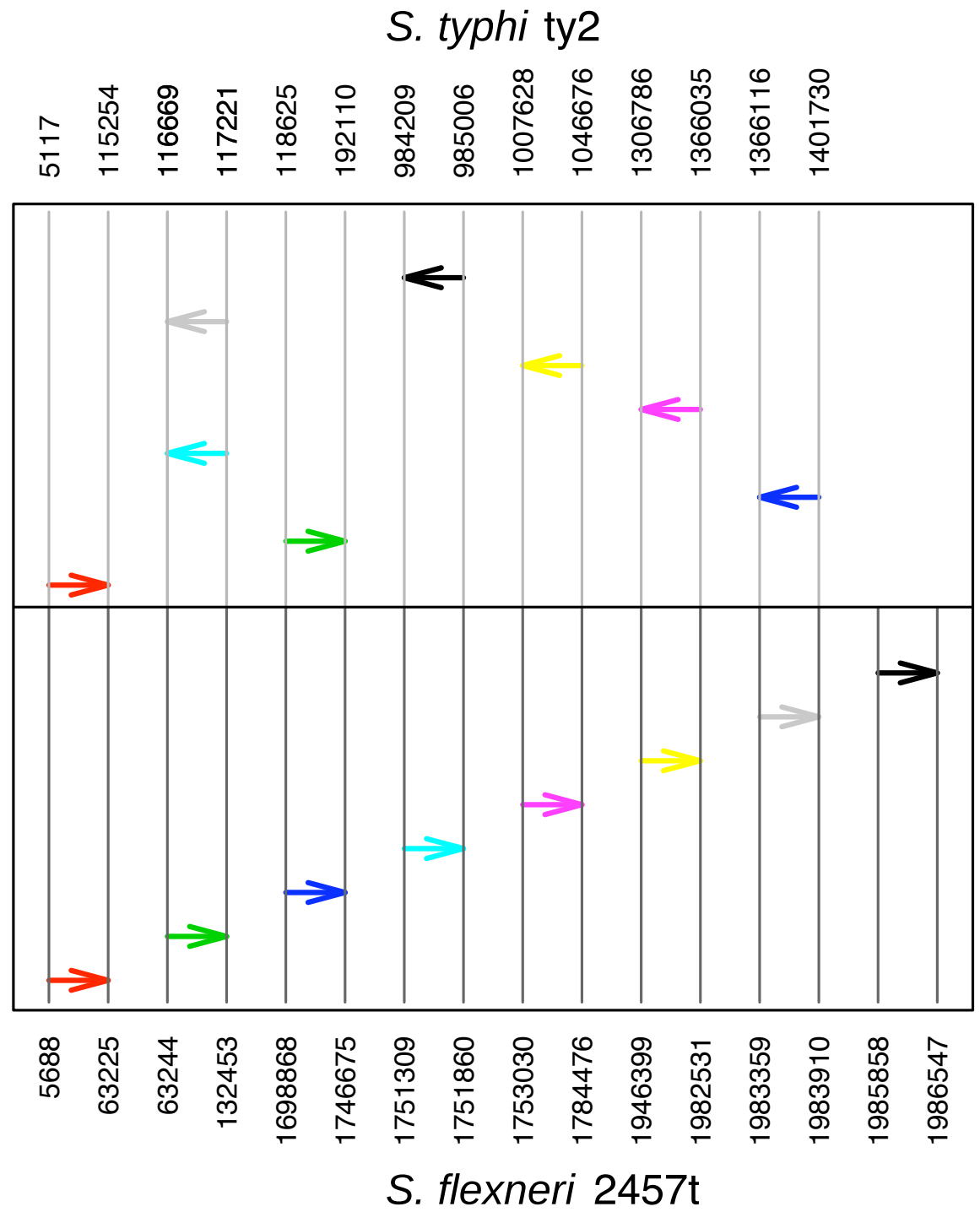

An example of a final conflict in the comparison of *S. flexneri* 2457t and *S. typhi* ty2. The cyan and the gray entries correspond to the same segment in *S. typhi* ty2. However, their corresponding segments in *S. flexneri* 2457t are different. Since these two entries are not collinear to any other entry, one cannot infer positional ortholog and inparalog relations based on length considerations. Hence, these two entries are considered a conflict.
