# Supplementary material for: An Integrative Method for Accurate Comparative Genome Mapping
Source: PLoS Comput Biol. 2006 Aug 4;2(8):e75. doi: 10.1371/journal.pcbi.0020075 (PMC1526463; doi:10.1371/journal.pcbi.0020075)
Supplement: Supplementary file 3 [file pcbi.0020075.sg002.pdf]

Figure S2

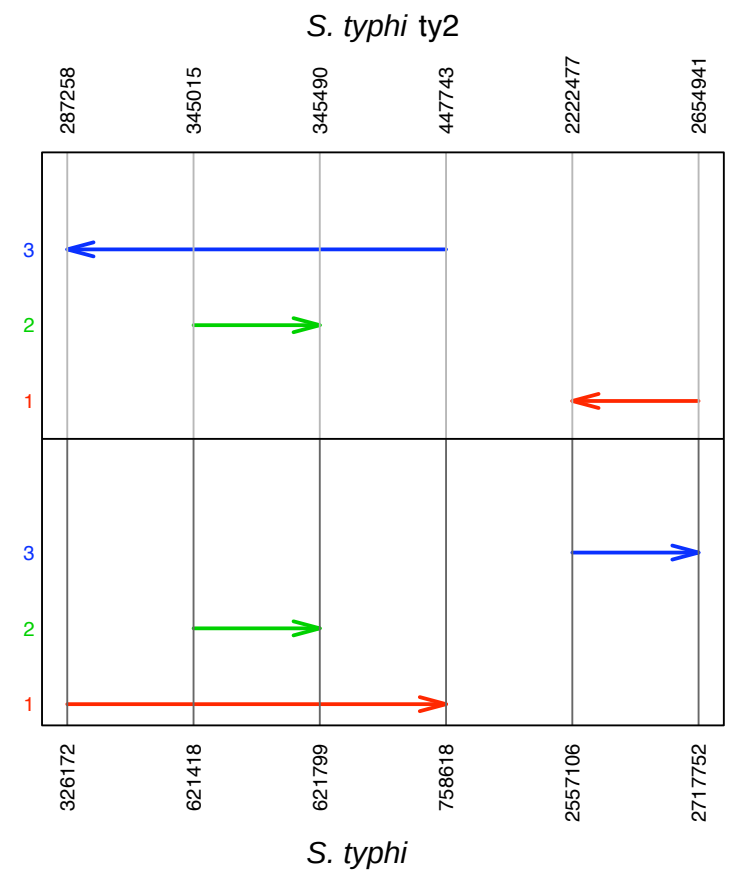

An example of a false KO anchor resulting from a nuisance cross overlap in the comparison of *S. typhi* ct18 and *S. typhi* ty2. Entry 2 (green) corresponds to K00357. By comparing it to Entries 1 and 3, it is easy to see that Entry 2 corresponds to a false anchor and is a nuisance cross overlap.
