# Supplementary material for: An Integrative Method for Accurate Comparative Genome Mapping
Source: PLoS Comput Biol. 2006 Aug 4;2(8):e75. doi: 10.1371/journal.pcbi.0020075 (PMC1526463; doi:10.1371/journal.pcbi.0020075)
Supplement: Supplementary file 4 [file pcbi.0020075.st001.pdf]

Table S1

Running Mauve with default parameters on the 10 prokaryotic pairs as in Table 2 — (a) and (b) — and on the example run — (c). Size: genome size. LCB(#/cov.): #: number of locally-collinear blocks. cov.: coverage of LCB blocks (Mauve's LCBs are comparable with MAGIC's RFs). Backbone: coverage of backbone segments (Mauve's Backbones are comparable with MAGIC's positional orthologs). Identity: mean identity of all backbone segments Uni.: the result of the Rao uniformity test with 5% as critical value — ✓accepting, - rejecting, and NA not applicable. The default value of some of the parameters are: The seed for calculating multi-MUMS equals $seed\_size = \log_2(\frac{g_1+g_2}{2})/1.5$ (for the given genomes the $seed\_size$ ranges between 12.8 and 15). The threshold for the gaps that are extracted from the alignments of collinear blocks for defining the "backbones" equals $maxBackboneGapSize = 50$, and the threshold for the length of backbone segments equals $minBackboneSize = 50$.

(a)

Organism	Size	LCB(#/cov.)	Backbone	Identity	Uni.
B. bronchiseptica	5339179	210/0.75	0.69	0.99	-
B. pertussis	4086189	210/0.95	0.91	0.99	-
H. pylori	1667867	100/0.84	0.79	0.94	-
H. pylori j99	1643831	100/0.85	0.80	0.94	-
N. meningitidis a	2184406	248/0.92	0.89	0.97	-
N. meningitidis b	2272351	248/0.90	0.85	0.97	-
S. typhi ty2	4791961	104/0.93	0.90	0.98	-
S. typhimurium	4857432	104/0.94	0.89	0.98	-
Y. pestis	4653728	124/0.97	0.94	0.99	-
Y. pseudotuberculosis	4744671	124/0.96	0.92	0.99	-

(b)

Organism	Size	LCB(#/cov.)	Backbone	Identity	Uni.
B. aphidicola aps	640681	10/0.99	0.95	0.77	✓
B. aphidicola sg	641454	10/0.99	0.95	0.77	✓
E. coli mg1655	4639675	151/0.91	0.84	0.98	-
S. flexneri 2457t	4599354	151/0.92	0.84	0.98	-
L. monocytogenes egd-e	2944528	82/0.98	0.89	0.88	-
L. innocua	3011208	82/0.92	0.87	0.88	-
P. abyssi	1765118	80/0.37	0.26	0.75	-
P. horikoshii	1738505	80/0.30	0.26	0.75	-
S. pyogenes m18	1895017	128/0.43	0.39	0.98	-
S. pyogenes ssi1	1894275	128/0.43	0.39	0.98	-

(c)

Organism	Size	LCB(#/cov.)	Backbone	Identity	Uni.
S. flexneri 2457t	4599354	283/0.84	0.68	0.80	-
S. typhi ty2	4791961	283/0.81	0.65	0.80	-
