# Supplementary material for: An Integrative Method for Accurate Comparative Genome Mapping
Source: PLoS Comput Biol. 2006 Aug 4;2(8):e75. doi: 10.1371/journal.pcbi.0020075 (PMC1526463; doi:10.1371/journal.pcbi.0020075)
Supplement: Supplementary file 5 [file pcbi.0020075.st002.pdf]

Table S2

Running Mauve with non-default parameters on the 10 prokaryotic pairs as in Table 2— (a) and (b) — and on the example run — (c). Size: genome size. LCB(#/cov.): #: number of locally-collinear blocks. cov.: coverage of LCB blocks (Mauve's LCB are comparable with MAGIC's RFs). Backbone: coverage of backbone segments (Mauve's Backbones are comparable with MAGIC's Positional orthologs). Identity: mean identity of all backbone segments. Uni.: the result of the Rao uniformity test with 5% as critical value — ✓accepting, - rejecting, and NA not applicable. The values of the changed parameters: The seed for calculating multi-MUMS is set to $seed\_size = 11$ (the value used in BLAST — MAGIC's local alignment engine). The threshold for the gaps that are extracted from the alignments of collinear blocks for defining the "backbones" is set to $maxBackboneGapSize = 200$ (analogous to $gapExtractLen$ used in Step 3 of MAGIC's preprocessing phase), and the threshold for the length of backbone segments is set to $minBackboneSize = 200$.

(a)

Organism	Size	LCB(#/cov.)	Backbone	Identity	Uni.
B. bronchiseptica	5339179	208/0.74	0.69	0.99	-
B. pertussis	4086189	208/0.95	0.91	0.99	-
H. pylori	1667867	134/0.93	0.91	0.94	-
H. pylori j99	1643831	134/0.94	0.92	0.94	-
N. meningitidis a	2184406	264/0.93	0.90	0.97	-
N. meningitidis b	2272351	264/0.92	0.87	0.97	-
S. typhi ty2	4791961	146/0.93	0.90	0.98	-
S. typhimurium	4857432	146/0.93	0.89	0.98	-
Y. pestis	4653728	123/0.97	0.94	0.99	-
Y. pseudotuberculosis	4744671	123/0.96	0.92	0.99	-

(b)

Organism	Size	LCB(#/cov.)	Backbone	Identity	Uni.
B. aphidicola aps	640681	90/0.97	0.95	0.75	✓
B. aphidicola sg	641454	90/0.97	0.95	0.75	✓
E. coli mg1655	4639675	188/0.91	0.84	0.98	-
S. flexneri 2457t	4599354	188/0.92	0.85	0.98	-
L. monocytogenes egd-e	2944528	151/0.96	0.90	0.88	-
L. innocua	3011208	151/0.91	0.87	0.88	-
P. abyssi	1765118	307/0.84	0.75	0.73	-
P. horikoshii	1738505	307/0.81	0.75	0.73	-
S. pyogenes m18	1895017	147/0.95	0.92	0.98	-
S. pyogenes ssi1	1894275	147/0.94	0.92	0.98	-

(c)

Organism	Size	LCB(#/cov.)	Backbone	Identity	Uni.
S. flexneri 2457t	4599354	537/0.81	0.68	0.79	-
S. typhi ty2	4791961	537/0.75	0.66	0.79	-
