# Supplementary material for: An Integrative Method for Accurate Comparative Genome Mapping
Source: PLoS Comput Biol. 2006 Aug 4;2(8):e75. doi: 10.1371/journal.pcbi.0020075 (PMC1526463; doi:10.1371/journal.pcbi.0020075)
Supplement: Supplementary file 6 [file pcbi.0020075.st003.pdf]

Table S3

Comparing coverages between MAGIC's (MG) results (Table 2 and example run) and Mauve's (MV) results when run with non-default parameters (Table S2). Differences in RFs coverage: The genomic portion covered by Mauve's LCBs but not by MAGIC's RFs (MV-MG), and vice versa (MG-MV). Differences in PO: The genomic portion covered by Mauve's Backbones but not by MAGIC's Positional orthologs (MV-MG), and vice versa (MG-MV). Differences in PS (Positional orthologs & Selfish): The genomic portion covered by Mauve's Backbones but not by MAGIC's +Prophages column, *i.e.*, after adding Inparalogs, Transposable elements and Prophages to Positional orthologs (MV-MG), and vice versa (MG-MV). Compare to Table 3. Pairs in (a) and (b) are as in Table 2. (c) contains the results for the example run.

(a)

Organism	Differences in RFs		Differences in PO		Differences in PS	
	MV-MG	MG-MV	MV-MG	MG-MV	MV-MG	MG-MV
B. bronchiseptica	0.033	0.0300	0.039	0.0046	0.036	0.036
B. pertussis	0.043	0.0075	0.048	0.0048	0.043	0.033
H. pylori	0.0031	0.032	0.017	0.023	0.014	0.040
H. pylori j99	0.0047	0.030	0.017	0.023	0.015	0.028
N. meningitidis a	0.0039	0.066	0.052	0.021	0.033	0.068
N. meningitidis b	0.0150	0.050	0.052	0.025	0.029	0.066
S. typhi ty2	0.0076	0.045	0.059	0.0090	0.045	0.044
S. typhimurium	0.0025	0.064	0.061	0.0096	0.046	0.048
Y. pestis	0.013	0.018	0.045	0.0041	0.027	0.051
Y. pseudotuberculosis	0.012	0.041	0.045	0.0070	0.040	0.016

(b)

Organism	Differences in RFs		Differences in PO		Differences in PS	
	MV-MG	MG-MV	MV-MG	MG-MV	MV-MG	MG-MV
B. aphidicola aps	0.064	0.027	0.079	0.028	0.077	0.028
B. aphidicola sg	0.064	0.028	0.083	0.026	0.082	0.027
E. coli mg1655	0.0094	0.046	0.060	0.0024	0.035	0.039
S. flexneri 2457t	0.0110	0.026	0.057	0.0026	0.033	0.099
L. monocytogenes egd-e	0.0026	0.034	0.063	0.0081	0.046	0.013
L. innocua	0.0120	0.084	0.058	0.0100	0.038	0.064
P. abyssi	0.011	0.053	0.038	0.035	0.037	0.040
P. horikoshii	0.013	0.069	0.030	0.034	0.030	0.035
S. pyogenes m18	0.013	0.030	0.15	0.0035	0.077	0.048
S. pyogenes ssi1	0.038	0.048	0.14	0.0016	0.017	0.060

(c)

Organism	Differences in RFs		Differences in PO		Differences in PS	
	MV-MG	MG-MV	MV-MG	MG-MV	MV-MG	MG-MV
S. flexneri 2457t	0.026	0.064	0.067	0.028	0.037	0.150
S. typhi ty2	0.020	0.110	0.066	0.026	0.057	0.074
