# Supplementary material for: An Integrative Method for Accurate Comparative Genome Mapping
Source: PLoS Comput Biol. 2006 Aug 4;2(8):e75. doi: 10.1371/journal.pcbi.0020075 (PMC1526463; doi:10.1371/journal.pcbi.0020075)
Supplement: Supplementary file 7 [file pcbi.0020075.st004.pdf]

Table S4

Running MAGIC while taking Mauve's backbone results as anchors. Size: genome size. RF(#/cov.): #: number of RF segments. cov.: coverage of RF segments. PO: coverage of positional orthologous segments. +IP: adding inparalogs to the previous column. +Transposable: adding transposable elements to the previous column. +Prophages.: adding prophages as well as phagic elements to the previous column. Identity: mean identity of all positional orthologs. Uni.: the result of the Rao uniformity test with 5% as critical value — ✓accepting, - rejecting, and NA not applicable. Compare to Table 2. * indicates that no prophage annotation was available for those species. Pairs in (a) and (b) are as in Table 2. (c) contains the results for the example run.

(a)

Organism	Size	RF(#/cov.)	PO	+IP	+Mobile DNA	+Prophages	Identity	Uni.
B. bronchiseptica	5339179	154/0.77	0.69	0.72	0.73	0.73	0.98	✓
B. pertussis	4086189	154/0.94	0.90	0.91	0.93	0.93	0.98	✓
H. pylori	1667867	38/0.96	0.92	0.92	0.93	0.93	0.93	-
H. pylori j99	1643831	38/0.97	0.93	0.94	0.94	0.94	0.93	-
N. meningitidis a	2184406	43/0.95	0.86	0.89	0.91	0.93	0.96	-
N. meningitidis b	2272351	43/0.90	0.83	0.86	0.87	0.89	0.96	-
S. typhi ty2	4791961	16/0.96	0.87	0.88	0.89	0.92	0.98	✓
S. typhimurium	4857432	16/1	0.86	0.86	0.87	0.91	0.98	✓
Y. pestis	4653728	43/0.97	0.90	0.90	0.94	0.97	0.98	✓
Y. pseudotuberculosis	4744671	43/0.98	0.89	0.89	0.90	0.90	0.98	✓

(b)

Organism	Size	RF(#/cov.)	PO	+IP	+Mobile DNA	+Prophages	Identity	Uni.
B. aphidicola aps	640681	1/0.96	0.93	0.93	0.93	0.93	0.75	NA
B. aphidicola sg	641454	1/0.96	0.93	0.93	0.93	0.93	0.75	NA
E. coli mg1655	4639675	37/0.95	0.80	0.80	0.83	0.86	0.97	✓
S. flexneri 2457t	4599354	37/0.93	0.80	0.80	0.88	0.92	0.98	-
L. monocytogenes egd-e	2944528	17/0.99	0.85	0.85	0.85	0.88	0.87	-
L. innocua	3011208	17/0.96	0.83	0.84	0.84	0.91	0.87	-
P. abyssi	1765118	88/0.89	0.74	0.74	0.74	0.74	0.73	-
P. horikoshii	1738505	88/0.88	0.75	0.75	0.75	0.75	0.73	-
S. pyogenes m18	1895017	9/0.96	0.72	0.73	0.73	0.84	0.98	✓
S. pyogenes ssi1	1894275	9/0.94	0.73	0.73	0.73	0.91	0.98	✓

(c)

Organism	Size	RF(#/cov.)	PO	+IP	+Mobile DNA	+Prophages	Identity	Uni.
S. flexneri 2457t	4599354	104/0.85	0.64	0.65	0.75	0.79	0.79	-
S. typhi ty2	4791961	104/0.85	0.62	0.62	0.63	0.68	0.79	-
