# Supplementary material for: An Integrative Method for Accurate Comparative Genome Mapping
Source: PLoS Comput Biol. 2006 Aug 4;2(8):e75. doi: 10.1371/journal.pcbi.0020075 (PMC1526463; doi:10.1371/journal.pcbi.0020075)
Supplement: Supplementary file 8 [file pcbi.0020075.st005.pdf]

Table S5

The one-to-one RF table resulting from running MAGIC mapping phase and filtering entries of length smaller than $1,000,000$ on the human-mouse chromosome X based on Blastz [12] chained output as available on UCSC Genome Browser [88].

Human start	Human end	Human length	Mouse start	Mouse end	Mouse length	Relative orientation
10227017	23348357	13121341	150203850	164763696	14559847	-
23666963	37056699	13389737	73857342	89765185	15907844	-
37172328	46883793	9711466	7607865	19168557	11560693	+
48121741	51179489	3057749	4434617	6640987	2206371	-
52959550	54945113	1985564	145286858	147128844	1841987	-
62764225	114341839	51577615	90503075	142261509	51758435	+
114916468	154057927	39141460	19817790	70965293	51147504	+
